# Stability analysis of landslide dam slopes based on the local strength reduction–random finite element method

**DOI:** 10.1371/journal.pone.0356280

**Published:** 2026-08-20

**Authors:** Ning Shi, Xiaohui Zhao, Jian Huang, Hongxing Li

**Affiliations:** Northwest Electrical Power Design Institute Co., Ltd. of China Power Engineering Consulting Group, Xi’an, People’s Republic of China; China University of Mining and Technology, CHINA

## Abstract

Landslide dams represent a critical geological hazard where rapid and accurate stability assessment is vital for emergency management. Following its formation, localized slope instability frequently triggers accelerated dam failure. Using the Tangjiashan landslide dam as a case study, this study utilizes a random finite element method within a Monte Carlo framework to establish a global random field model. The research quantitatively evaluates how shear strength parameter variability in distinct sub-zones affects the safety factor and failure probability, employing the shear strength reduction technique. Furthermore, a local random field model based on the local strength reduction method was developed. Validation against the global model confirms its feasibility and computational efficiency. These findings offer a scientific basis and technical support for landslide dam stability analysis.

## 1. Introduction

A landslide dam is a natural blockage of a river channel caused by debris accumulation triggered by rainfall, earthquakes, or anthropogenic activities [1]. These dams represent severe geological hazards that pose significant risks to life, property, and social stability [2,3]. China experiences one of the highest frequencies of landslide dam formation globally [4]. In contrast to engineered dams, natural landslide dams comprise heterogeneous mixtures of materials—ranging from clay and sand to gravel and rock fragments—with particle sizes spanning micrometers to meters [5]. Furthermore, the pronounced spatial variability of these materials creates substantial uncertainty in slope stability assessments [6–8]. Despite this, advanced technical support for the rapid risk assessment of landslide dams remains inadequate in China, which fails to meet current emergency management demands [9]. Consequently, the rapid evaluation of landslide dam slope stability is not only the foundational step for risk assessment but also an urgent imperative for disaster prevention and mitigation.

Significant attention has been devoted to the anti-sliding stability of landslide dam slopes. Similarly, Mizuyama et al. [10] applied the simplified Bishop’s method to the Taka-iso-yama landslide dam in Japan; by accounting for geometry, material properties, and seepage, their computed results showed good agreement with field observations. Focusing on sliding failure mechanisms, Awal et al. [11] used a three-dimensional Janbu method incorporating seepage effects. Their numerical simulations aligned closely with experimental data regarding pore-water movement, critical slip surface location, and failure time. Addressing the combined effects of rapid drawdown and seismic loading, Song [12] employed the finite element strength reduction method for the Tianchi landslide dam. The study concluded that seismic loading exerts a significantly greater influence on slope stability than rapid lake-level drawdown. Hu et al. [13] used the Swedish slice method to analyze the Tangjiashan landslide dam, evaluating upstream and downstream stability under varying aftershock intensities and water levels while examining potential breach mechanisms. Furthermore, Luo [14] incorporated coupled seepage–stress–strain behavior using GeoStudio to assess the Tangjiashan landslide dam, finding that while localized failure is possible, the overall structure remains stable. He et al. [15] similarly applied GeoStudio to the Dongcuoqu glacial lake landslide dam, concluding that its stability is satisfactory. Although these deterministic numerical analyses provide valuable site-specific references, they generally fail to account explicitly for the inherent variability of material parameters. Additionally, the simplification of actual dam geometry in some studies may compromise the accuracy and reliability of stability predictions.

To comprehensively capture the spatial variability of landslide dam materials and deliver scientifically sound, timely support for emergency decision-making, this study proposes a novel slope stability analysis framework integrating random finite element modeling with local strength reduction. Initially, Python-based scripts are developed to discretize spatial random fields of dam material strength parameters via Cholesky decomposition. Within a Monte Carlo simulation framework, a global random field model of the dam slope is established using finite element software. The shear strength reduction method is then employed to quantify the impacts of strength parameter autocorrelation, coefficient of variation (COV), and cross-correlation on the safety factor (*F*_*s*_) and failure probability. To facilitate rapid stability computation under material parameter uncertainty, a local random field model is constructed by focusing on elements within the potential slip zone, which is subsequently used to analyze the effects of strength parameter variability on the *F*_*s*_ and failure probability. Finally, the feasibility and computational efficiency of the local random field model are rigorously validated against the global random field model, providing a robust and efficient basis for the rapid risk assessment of landslide dams.

## 2. Method

### 2.1. Random finite element method

The random finite element method (RFEM) approximates the continuous fluctuation of random fields by generating spatially correlated random variables over the spatial domain. These variables are subsequently mapped onto the finite element mesh for analysis. Here, strength parameters are discretized as random fields using the midpoint method based on Cholesky decomposition. The key steps for this discretization, incorporating parameter heterogeneity, autocorrelation, and cross-correlation, are as follows:

The random field elements correspond one-to-one with those of the finite element mesh. The autocorrelation matrix ***C***_*n×n*_ is formulated using the centroid coordinates of the random field elements, after which Cholesky decomposition is employed to factorize the matrix into the product of a lower triangular matrix and its transpose, i.e.,


$$\begin{array}{c} C_{n\times n}=L_{\text{1}}L_{\text{1}}^{T} \end{array}$$ (1)

Regarding the strength parameters cohesion (*c*) and internal friction angle (*φ*) of the landslide dam material, the n-dimensional standard normal random vector ***S***_*n×2*_ that incorporates the cross-correlation structure is generated via Eqs. (2) and (3):


$$\begin{array}{c} S_{n\times 2}=L_{\text{1}}\xi_{n\times 2} \end{array}$$ (2)

$$\begin{array}{c} \xi_{n\times 2}=\left(\xi_{c},\;\xi_{\varphi }\right) \end{array}$$ (3)

In the formula (2) and formula (3), ***ξ***_*n×2*_ is an n-dimensional random vector of independent standard normal distributions of *c* and *φ*.

Using the Latin hypercube sampling technique, a random sample matrix ***ξ*** that satisfies the standard normal distribution is generated. The cross-correlation relationship between the material strength parameters *c* and *φ* is characterized by the cross-correlation matrix composed of the cross-correlation coefficients *A*_***ξ****c,****ξ****φ*_:


$$\begin{array}{c} {ℛ}_{2\times 2}=\left[\begin{array}{cc} @cc@1 & A_{\xi_{c},\xi_{\varphi }}\\ A_{\xi_{c},\xi_{\varphi }} & A_{\xi_{c},\xi_{\varphi }} \end{array}\right] \end{array}$$ (4)

The cross-correlation coefficient *A*_*ξc,ξφ*_ of the standard normal space and the cross-correlation coefficient *A′*_***ξ****c,****ξ****φ*_ of the original log-normal space are generally not equal. The process of converting from the standard normal distribution to the log-normal distribution is called the equiprobability transformation. The specific transformation relationship is shown in Eq. (5).


$$\begin{array}{c} A_{\xi_{c},\xi_{\varphi }}=\frac{ln\left(1+{A^{\prime }}_{\xi_{c},\xi_{\varphi }}COV_{\xi_{c}}COV_{\xi_{\varphi }}\right)}{\sqrt{ln\left(1+CO{V_{\xi_{c}}}^{\text{2}}\right)}\sqrt{ln\left(1+CO{V_{\xi_{\varphi }}}^{\text{2}}\right)}} \end{array}$$ (5)

From Eq. (5), it can be calculated that the *n*-dimensional standard normal distribution random vector that meets both the autocorrelation and cross-correlation requirements can be obtained:


$$\begin{array}{c} Z_{n\times 2}=L_{\text{1}}\xi {L_{\text{2}}}^{T} \end{array}$$ (6)

In the formula, ***L***_*2*_ is the lower triangular matrix obtained by performing the Cholesky decomposition on the cross-correlation matrix ***R***.

The log-normal distributed random vector ***α***_*n×2*_ = (***α***_*c*_, ***α***_*φ*_) that ultimately meets the correlation requirements can be obtained by taking the exponent of the corresponding normal distributed random vector, that is:


$$\begin{array}{c} \alpha_{i}=exp\left(\mu_{lni}+\sigma_{lni}Z_{i}\right),\;i=c,\;\varphi \end{array}$$ (7)

$$\begin{array}{c} \mu_{lni}=ln\mu_{i}-\frac{\text{1}}{\text{2}}\sigma_{lni}^{\text{2}} \end{array}$$ (8)

In the formula, *σ*_*i*_ represents the standard deviation of the log-normal variable *i*, *σ*_*lni*_ represents the standard deviation of the normal variable ln*i*, *μ*_*i*_ represents the mean value of the log-normal variable *i*, and *μ*_*lni*_ represents the mean value of the normal variable ln*i*.

### 2.2. Local strength reduction method

The local strength reduction method was proposed by Yang et al. [16]. It is an analysis method that gradually reduces the strength parameters *c* and *φ* of the soil in the potential sliding zone of the slope to make the slope reach the limit equilibrium state. This method improves the calculation efficiency while ensuring accuracy. The specific steps are as follows: Firstly, the sliding zone range is determined using the global strength reduction method, and the relevant soil units are extracted. Then, the calculation model is initialized, and only the strength parameters of the sliding zone soil are reduced, with the reduction method being the same as that of the global strength reduction method. The basic principle of the global strength reduction method is to divide the tangent values of the strength parameters *c* and *φ* by a reduction coefficient *F*_*s*_ in the finite element software, obtaining a set of new *c′*, tan*φ′* values. The reduced shear strength parameters are *c′* and *φ′*, and the corresponding calculation methods are shown in Eqs. (9) and (10). Then, *c′* and *φ′* are input as new strength parameters, and the finite element calculation is performed. When the calculation does not converge, the corresponding reduction factor Fs is defined as the factor of safety (*F*_*s*_) of the slope.


$$\begin{array}{c} c^{\prime }=c/F_{s} \end{array}$$ (9)

$$\begin{array}{c} \varphi^{\prime }=arctan(tan\varphi /F_{s}) \end{array}$$ (10)

## 3. Deterministic stability analysis of the tangjiashan landslide dam

### 3.1. Overview of the Tangjiashan landslide dam

**Fig 1:**
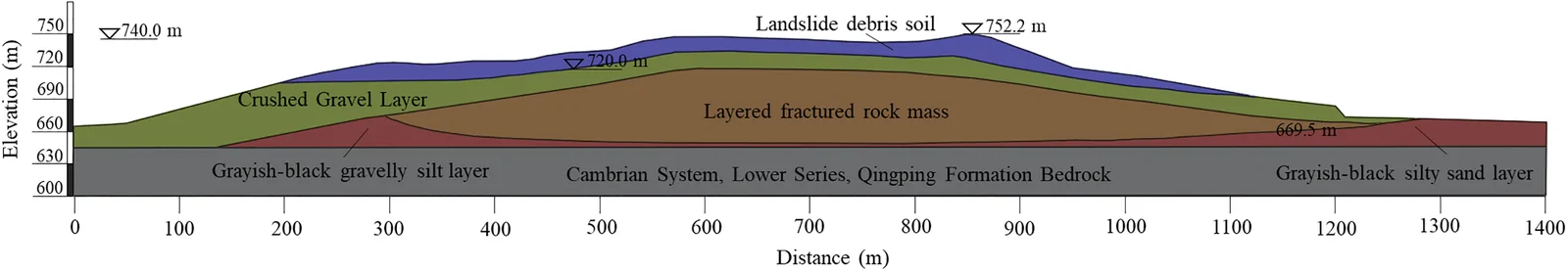
Typical cross-section diagram of Tangjiashan landslide dam.

The case study focuses on the Tangjiashan landslide dam [17], located approximately 4 km upstream of Qushan Town in the former Beichuan County seat, Sichuan Province, China. Triggered by the 2008 Wenchuan earthquake, the dam’s cross-sectional profile and dimensions are illustrated in Fig 1 [18]. Characterized as a high-velocity landslide deposit, the dam exhibits distinct elevation differences across its profile: the forebulge reaches a maximum elevation of 793.9 m, whereas the rear bulge has a minimum surface elevation of 752.2 m [14]. As depicted in Fig 1, the stratigraphic sequence from the base to the surface consists of pseudo-bedded fractured rock (derived from the disintegration of weakly weathered bedrock), a blocky gravel and rubble layer (resulting from the fragmentation of strongly weathered rock), and gravelly soil (originating from the surficial yellowish-brown slope layer). For the purpose of material zoning, the gravelly soil, blocky gravel, pseudo-bedded fractured rock, gray-black silty clay, and bedrock zones are designated as *D*_*g*_, *D*_*b*_, *D*_*r*_, *D*_*s*_, and *D*_*f*_, respectively.

### 3.2. Deterministic analysis of landslide dam slope stability

This section evaluates the slope stability of the Tangjiashan landslide dam under long-term rainfall conditions, utilizing saturated-state strength parameters for the dam materials. The physical and mechanical parameter values assigned to the dam materials are based on field investigations conducted by Hu et al. [18]. Furthermore, the degradation patterns of strength parameters for coarse-grained soils under saturation, as proposed by Wang et al. [19] have been incorporated. Consequently, the final strength parameters for the distinct material zones used in this study are derived from these sources and are summarized in Table 1.

**Table 1:** Material parameters of Tangjiashan landslide dam under saturated condition.

Material Properties	Density (g/cm^3^)	*c* (kPa)	*φ* (º)	Elastic modulus (kPa)	Poisson’s ratio
***D***_***g***_	1.9	7.9	18.04	1.8e5	0.4
***D***_***b***_	2.1	23.7	28.7	8e5	0.2
***D***_***r***_	2.2	36.6	41.265	5e6	0.15
***D***_***s***_	1.9	8.5	18	5e4	0.23
***D***_***f***_	2.61	3800	51.58	2e7	0.25

Following the formation of the Tangjiashan landslide dam, the observed rate of lake water level rise was 2.64 m/d. For the stability analysis of the dam slopes, the water level was simulated to rise from the dry season elevation of 665.0 m to 752.2 m. Consequently, eleven analysis steps are employed to simulate the progressive increase in hydrostatic load, representing the gradual rise in water level.

The boundary conditions for the finite element model of the Tangjiashan landslide dam are defined as follows: roller constraints are applied to the lateral boundaries, fixed constraints to the base, hydrostatic pressure to the upstream face, and gravity loads globally. The materials in different zones of the dam are modeled using an ideal elastic-plastic constitutive model based on the Mohr-Coulomb yield criterion.

**Fig 2:**
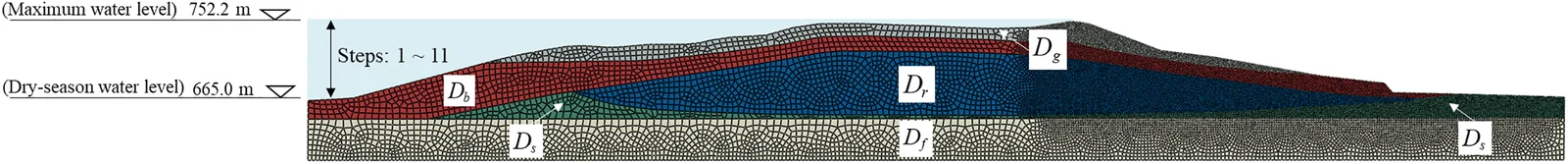
Finite element calculation model of Tangjiashan landslide dam.

To determine the appropriate mesh size, a systematic convergence analysis was performed on the downstream slope zone using a progressive mesh refinement approach. The safety factor *F*_*s*_ was adopted as the evaluation criterion, and the convergence criterion was predefined as follows: the mesh density is considered acceptable when the relative change in *F*_*s*_ between two successive mesh levels is less than 1%. The computational results show that when the mesh size in the downstream slope zone was refined from 4 m to 2 m, Fs increased from 1.152 to 1.156, representing a relative change of only 0.35%. Further refinement to 1 m yielded *F*_*s*_ = 1.157, with a relative change of merely 0.09% compared with the 2 m mesh. Considering both computational accuracy and efficiency, the final mesh was designed with a 2 m element size in the downstream slope zone and a 4 m element size elsewhere. The finite element mesh is illustrated in Fig 2. This mesh scheme comprises a total of 16,169 nodes and 15,768 elements.

**Fig 3:**
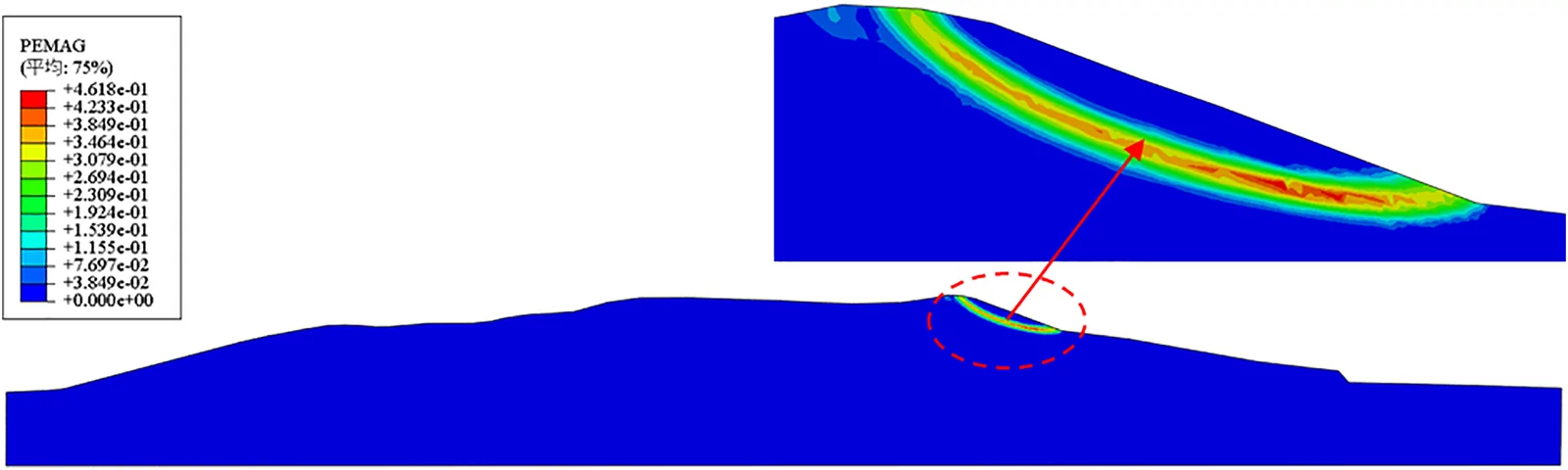
Distribution of equivalent plastic strain.

Applying the global element strength reduction method, the equivalent plastic strain contours illustrating the failure of the Tangjiashan landslide dam slope are obtained, as shown in Fig 3. The results indicate that the plastic yield zone became fully connected within the *D*_*g*_ zone, forming a continuous slip surface extending from the crest to the toe on the downstream side. An examination of the elements revealed that those along the slip surface are situated entirely within the downstream gravel soil layer. In the stability analysis, the reduction coefficient (*F*_*s*_) corresponding to the formation of a continuous slip surface is 1.156. The *F*_*s*_ determined at the point of non-convergence is 1.159, which aligns well with the findings of Hu et al. [18].

## 4. Landslide dam slope stability analysis based on global random field model

### 4.1. Shear strength parameters of random materials

The material composition exhibits significant heterogeneity across the four distinct zones (*D*_*g*_, *D*_*b*_, *D*_*r*_, and *D*_*s*_) of the Tangjiashan landslide dam. Consequently, unique horizontal and vertical autocorrelation distances should be assigned to the strength parameters of each zone. Typically, the horizontal correlation length is greater than the vertical correlation length [20]. The cross-correlation coefficient ranges for the strength parameters *c* and *φ* are assumed to be consistent across all zones. The characteristic parameters for the random field model of the Tangjiashan landslide dam materials are presented in Table 2, with default values indicated in bold.

**Table 2:** Characteristic parameters of random field model of Tangjiashan landslide dam materials.

Zone	Parameter	Mean value	COV	Autocorrelation distance (m)	
				Horizontal	Vertical
***D***_***g***_	*c*_*g*_ (kPa)	7.9	0.1, **0.2**, 0.3, 0.5	20, 30, **50**, 70	0.5, 1, **2**, 3
	*φ*_*g*_ (º)	18.04	**0.1**, 0.15, 0.2, 0.25	20, 30, **50**, 70	0.5, 1, **2**, 3
	*A*_*c-g, φ-g*_	−0.7, **−0.5**, 0, 0.5			
***D***_***b***_	*c*_*b*_ (kPa)	23.7	0.1, **0.25**, 0.4, 0.7	25, 35, **55**, 75	1, 2, **4**, 8
	*φ*_*b*_ (º)	28.7	0.1, **0.15**, 0.2, 0.3	25, 35, **55**, 75	1, 2, **4**, 8
	*A*_*c-b, φ-b*_	−0.7, **−0.5**, 0, 0.5			
***D***_***r***_	*c*_*r*_ (kPa)	36.6	0.1, 0.3, **0.55**, 0.8	30, 40, **65**, 95	2, 4, **6**, 8
	*φ*_*r*_ (º)	41.265	0.15, 0.2, **0.25**, 0.35	30, 40, **65**, 95	2, 4, **6**, 8
	*A*_*c-r, φ-r*_	−0.7, **−0.5**, 0, 0.5			
***D***_***s***_	*c*_*s*_ (kPa)	8.5	0.1, **0.15**, 0.2, 0.3	15, 20, **30**, 50	0.2, 0.5, **1**, 2
	*φ*_*s*_ (º)	18	0.05, **0.1**, 0.15, 0.25	15, 20, **30**, 50	0.2, 0.5, **1**, 2
	*A*_*c-s, φ-s*_	−0.7, **−0.5**, 0, 0.5			

The determination of horizontal and vertical autocorrelation distances is intrinsically linked to the geotechnical properties of the rock and soil mass. Unlike engineered fills, the materials comprising the landslide dam are naturally deposited structures that have not undergone artificial compaction. Consequently, these materials exhibit a high degree of heterogeneity and are typically under-consolidated. Generally, the horizontal autocorrelation distance of shear strength parameters exceeds the vertical autocorrelation distance [21]. In general, soil structures with fewer pore spaces are more compact, resulting in tighter inter-particle connections; under such conditions, the autocorrelation distance is typically smaller [22]. This is attributed to the fact that in more compact soil structures, the variation in soil properties between adjacent observation points is more continuous, allowing spatial correlation to persist over longer distances, thereby resulting in a relatively smaller autocorrelation distance. Regarding the four material zones of the Tangjiashan landslide dam, the *D*_*s*_ zone possesses the most compact structure due to sedimentation processes; therefore, the horizontal and vertical autocorrelation distances are assigned smaller values. Conversely, the *D*_*r*_ and *D*_*b*_ zones consist of fractured rock blocks with significant structural porosity, necessitating larger autocorrelation distance values. The material in the *D*_*g*_ zone is primarily soil containing gravel with small pores; consequently, its autocorrelation distance is set to be greater than that of the *D*_*s*_ zone but smaller than that of the *D*_*r*_ and *D*_*b*_ zones. In summary, the horizontal and vertical autocorrelation distances for the shear strength parameters in different zones of the Tangjiashan landslide dam are defined in Table 2, and the influence of a broad range of autocorrelation distance values on dam slope stability is investigated.

### 4.2. Calculation procedure of random field model for landslide dam slope stability

**Fig 4:**
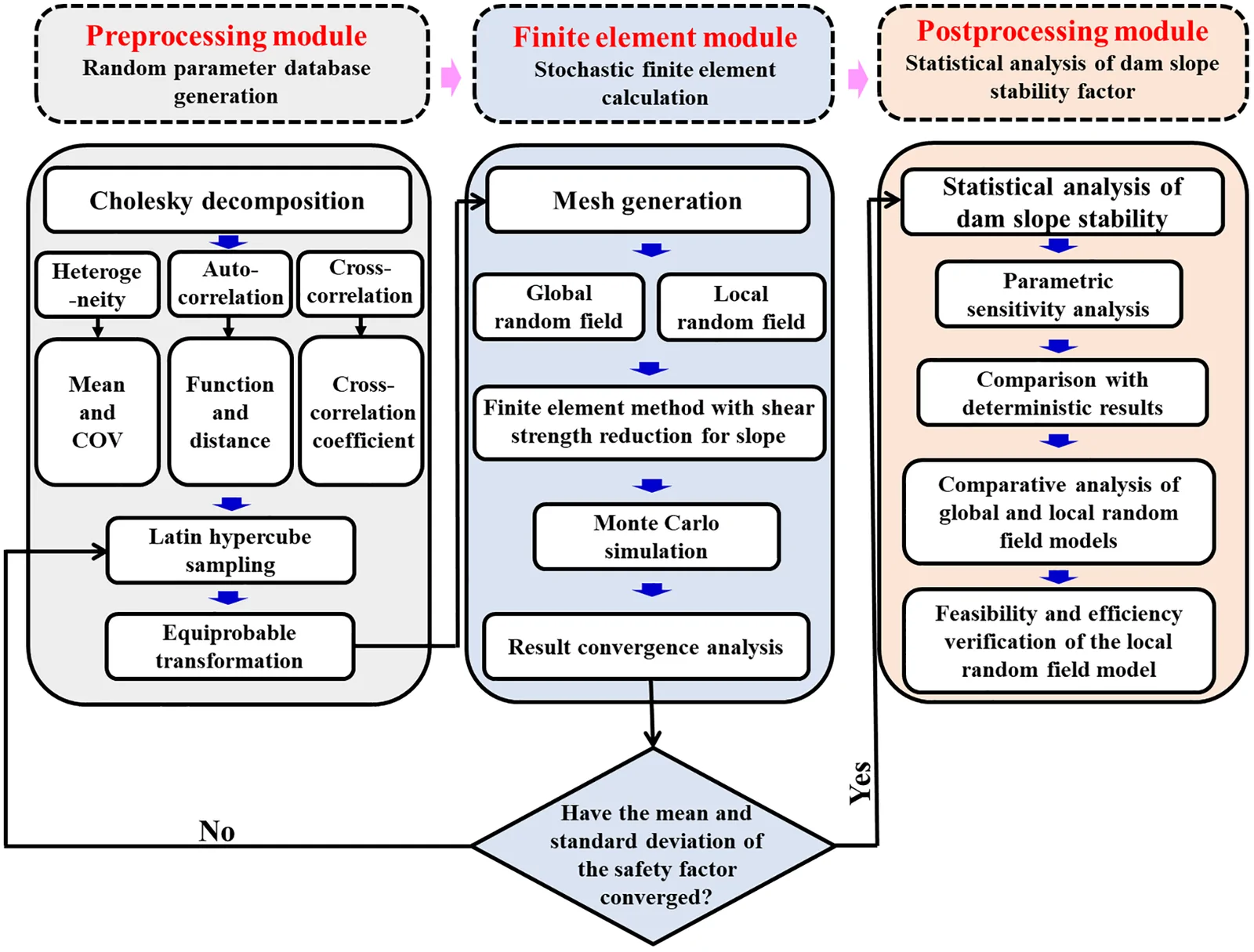
Landslide dam slope stability calculation flow chart.

This study implements the full workflow of the RFEM by combining Python programming with finite element software. As illustrated in Fig 4, the dam slope stability analysis based on the RFEM consists of three core modules: the pre-processing module, the finite element analysis module, and the post-processing module. Specifically, the pre-processing module focuses on generating the random parameter database, the finite element analysis module conducts random finite element calculations, and the post-processing module carries out statistical analysis of the dam slope stability *F*_*s*_.

In the preprocessing module, the first step is to determine the distribution characteristics of the shear strength parameters of the dam slope material, including the probability distribution form, mean value, COV, autocorrelation function, autocorrelation distance, and cross-correlation coefficient. Then, a Python program is developed, and the Karhunen-Loève decomposition method is employed to discretize the random field of the strength parameters. Finally, the Latin hypercube sampling technique and equal probability transformation methods are adopted to generate a random shear strength parameter database that conforms to the log-normal distribution. In the finite element analysis module, the first step is to divide the grid of the dam slope finite element model and assign the randomly generated material strength parameters to the finite element grid one by one. Then, within the framework of Monte Carlo simulation, the method of submitting inp type files in batches is used to establish the global random field model and the local random field model, and the finite element calculation of dam slope strength reduction is performed. Finally, it is determined whether the mean value and COV of the obtained *F*_*s*_ converge; if not, Latin hypercube sampling is conducted again until the results converge. In the post-processing module, the results of the random finite element calculations are extracted in batches, and parameter sensitivity statistical analysis is carried out. Then, the dam slope *F*_*s*_ obtained based on the random field model is compared with the deterministic result, and the *F*_*s*_, failure probability, and calculation efficiency calculated based on the global and local random field models are compared and analyzed. Finally, the feasibility and efficiency of the local random field model are verified.

### 4.3. Typical implementation of the global random field model

**Fig 5:**
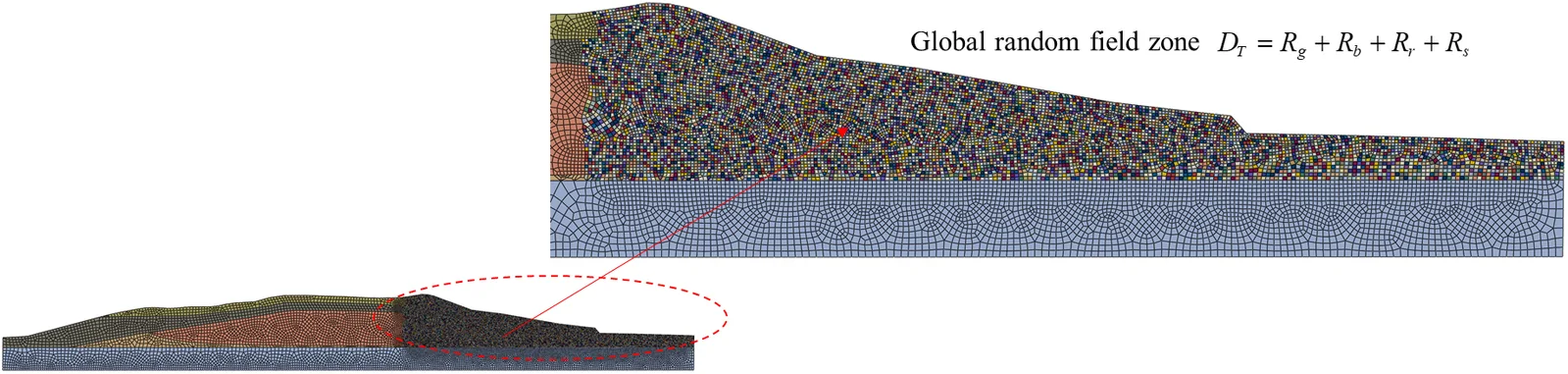
Global random zone selection.

Since the deterministic calculation results (Fig 3) show that the sliding failure surface of the Tangjiashan landslide dam is located on the downstream side of the dam slope, this paper selects the downstream portions of *D*_*g*_, *D*_*b*_, *D*_*r*_, and *D*_*s*_ as the global random field zones for material strength parameters. The global random field zone consists of the grid cells marked with various colors in the downstream part of the dam slope, as shown in Fig 5. In Fig 5, the random field zones of the four partitions are denoted as *R*_*g*_, *R*_*b*_, *R*_*r*_, and *R*_*s*_ respectively, and the sum of the four random field zones are denoted as *D*_*T*_. Since *D*_*T*_ represents the entire set of random field zones of the dam slope, the random field model based on the *D*_*T*_ zone is also referred to as the global random field model. In Fig 5, the *R*_*g*_ zone contains a total of 1074 grid cells, the *R*_*b*_ zone contains a total of 1935 grid cells, the *R*_*r*_ zone contains a total of 4118 grid cells, and the *R*_*s*_ zone contains a total of 1782 grid cells. This paper establishes a global random field model based on the 8909 grid cells of the four random field zones and conducts subsequent dam slope stability analysis.

**Fig 6:**
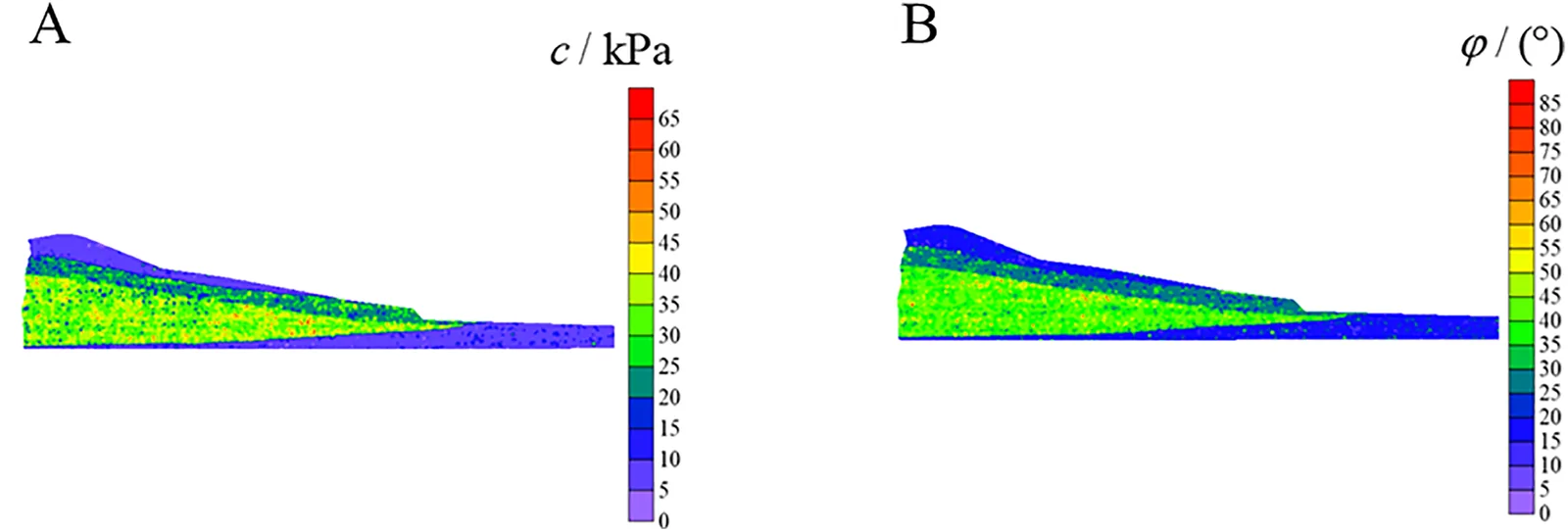
A typical realization of *c* and *φ* based on the global random field model. (A) *c.* (B) *φ.*

Taking a certain combination in Table 2 as an example, the contour plots of the shear strength parameters *c* and *φ* of the global random field model under a typical implementation are drawn, as shown in Fig 6. Fig 6A shows the contour plot of *c* under a typical implementation, and Fig 6B shows the contour plot of *φ* under a typical implementation. From Fig 6, it can be observed that the contour plots of the random parameters *c* and *φ* both exhibit certain horizontal band distribution characteristics, mainly because the horizontal autocorrelation distances of *c* and *φ* are greater than their vertical autocorrelation distances. Since the cross-correlation coefficient of *c* and *φ* under this typical random condition is −0.5, *c* and *φ* in Fig 6 also show a certain negative correlation as expected.

### 4.4. Results

#### 4.4.1. Convergence analysis of Monte Carlo simulation results.

**Fig 7:**
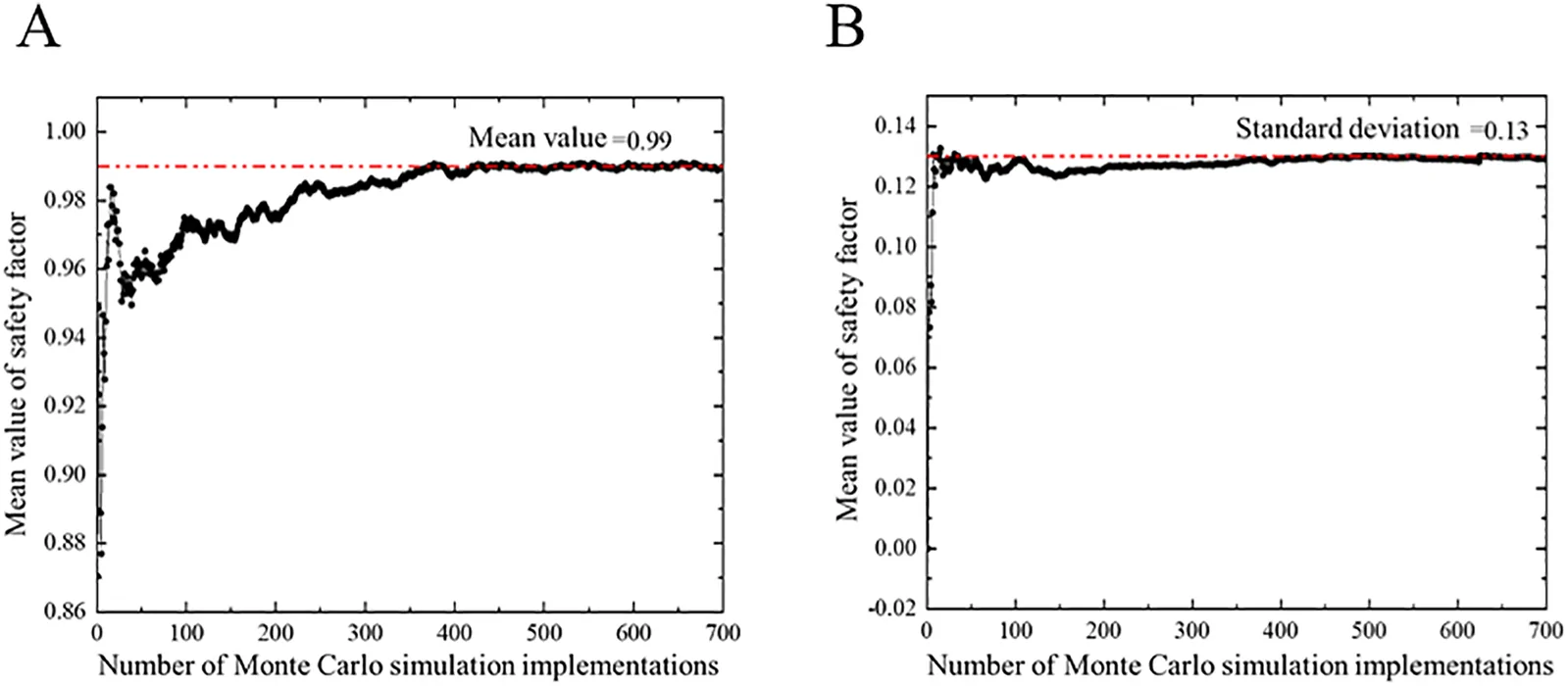
Convergence curve of *F*_*s*_ of landslide dam slope based on global random field mode. (A) Convergence curve of mean value of *F*_*s*_. (B) Convergence curve of standard deviation of *F*_*s*_.

Within the Monte Carlo simulation framework, sufficient repetitions are required to stabilize the trends of the mean value and standard deviation of the *F*_*s*_ [23]. To obtain stable statistical outputs of the *F*_*s*_, 700 Monte Carlo simulations are conducted for each parameter combination in Table 2 in this section. Fig 7 presents the calculation results of the *F*_*s*_ from 700 Monte Carlo simulations under Combination 1. Fig 7A and 7B are the convergence curves reflecting the changes in the mean value and standard deviation of the *F*_*s*_ with the variation in the number of Monte Carlo simulations. As can be seen from Fig 7, 500 simulations are already sufficient to ensure adequate convergence of the *F*_*s*_. In the subsequent analysis, 500 random simulations of material strength parameters are performed to ensure sufficiently reliable results are obtained with the minimum possible computational workload.

#### 4.4.2. The influence of horizontal autocorrelation distance.

**Fig 8:**
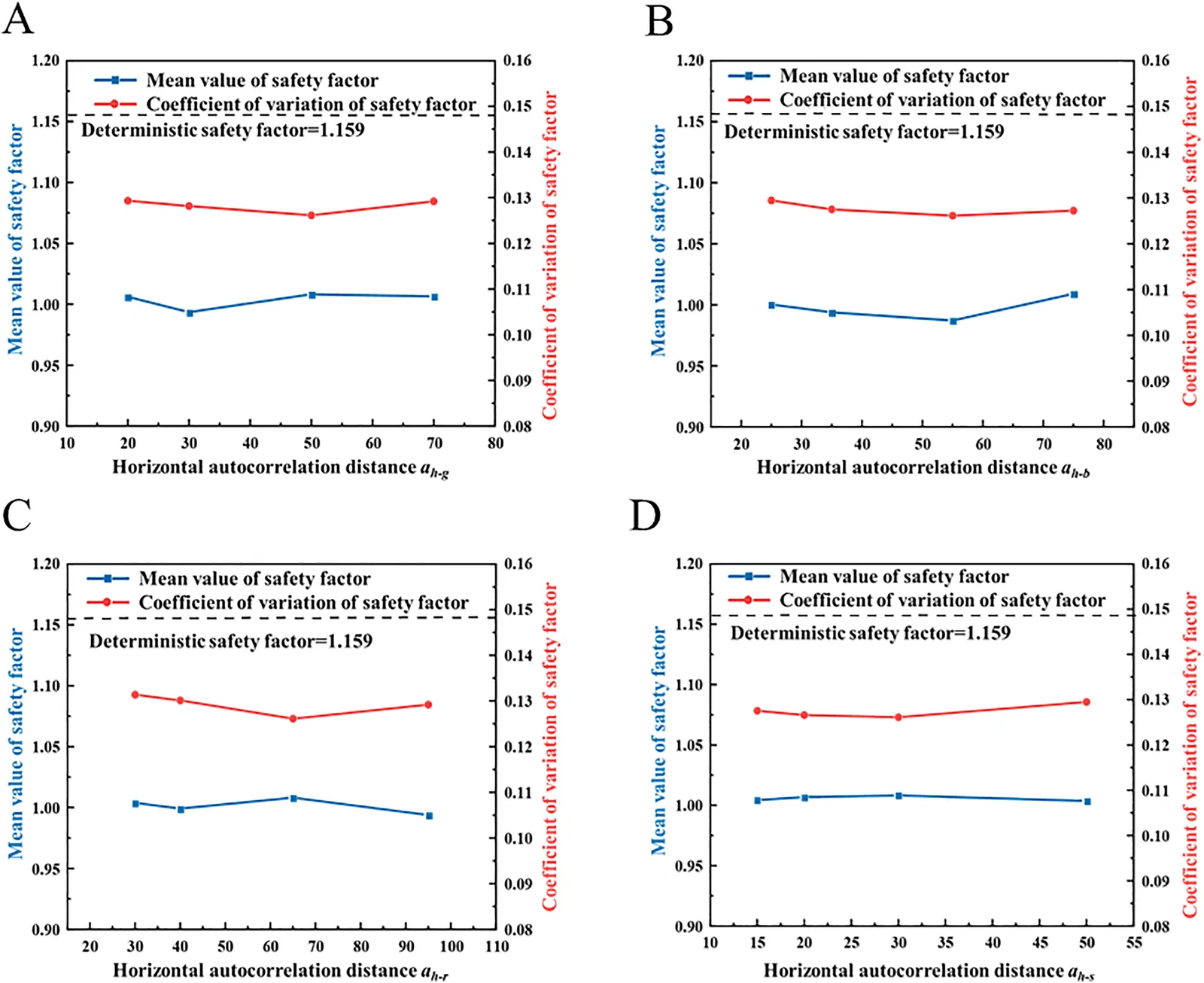
The influence of horizontal autocorrelation distance on the mean value and COV of *F*_*s*_. (A) *R*_*g*_ zone. (B) *R*_*b*_ zone. (C) *R*_*r*_ zone. (D) *R*_*s*_ zone.

Sections 4.4.2 to 4.4.6 adopt the mean value and COV of *F*_*s*_ calculated with a cross-correlation coefficient of –0.5. This section first analyzes how the mean value and COV vary with the horizontal autocorrelation distance, as shown in Fig 8. Compared with the deterministic *F*_*s*_, the *F*_*s*_ obtained by considering the horizontal autocorrelation distances of material strength parameters in different random field zones are consistently smaller. Across the considered horizontal autocorrelation distances, the maximum mean value of *F*_*s*_ is 1.009, and the minimum is 0.987. As the horizontal autocorrelation distances of the *D*_*g*_, *D*_*b*_, and *D*_*r*_ zones increase, the COV of the *F*_*s*_ first decreases and then increases. Across these distances, the maximum COV of the *F*_*s*_ is 0.131, and the minimum is 0.126. The mean value of *F*_*s*_ first decreases, then increases, and then decreases again as the horizontal autocorrelation distances of the *D*_*g*_ and *D*_*r*_ zones increase. The change in the horizontal autocorrelation distance of the *D*_*s*_ zone has the least significant impact on *F*_*s*_.

**Fig 9:**
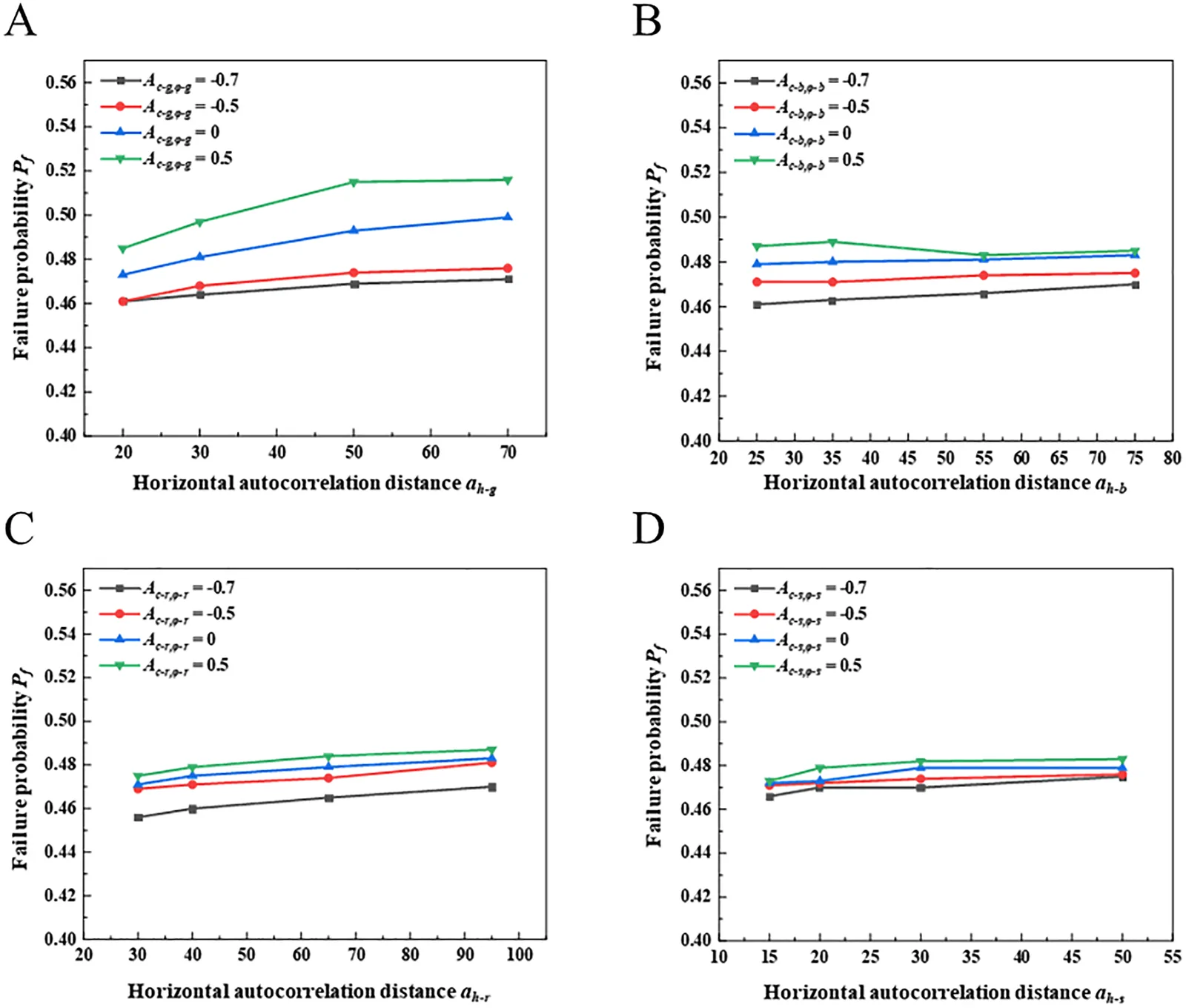
The influence of horizontal autocorrelation distance on failure probability. (A) *R*_*g*_ zone. (B) *R*_*b*_ zone. (C) *R*_*r*_ zone. (D) *R*_*s*_ zone.

Fig 9 illustrates the influence of the horizontal autocorrelation distance of material strength parameters in different random field zones on the failure probability of the dam slope. As shown in the Fig 9, the greater the cross-correlation coefficient of the materials across the four random field zones, the higher the failure probability. Furthermore, the larger the horizontal autocorrelation distance, the higher the failure probability of the dam slope. Across the range of horizontal autocorrelation distances considered, the maximum failure probability of the dam slope is 0.516, and the minimum is 0.456. Among the four random field zones, the material strength parameters in the *D*_*g*_ zone have the most significant influence on the failure probability, whereas those in the *D*_*s*_ zone exhibit no obvious influence.

#### 4.4.3. The influence of vertical autocorrelation distance.

**Fig 10:**
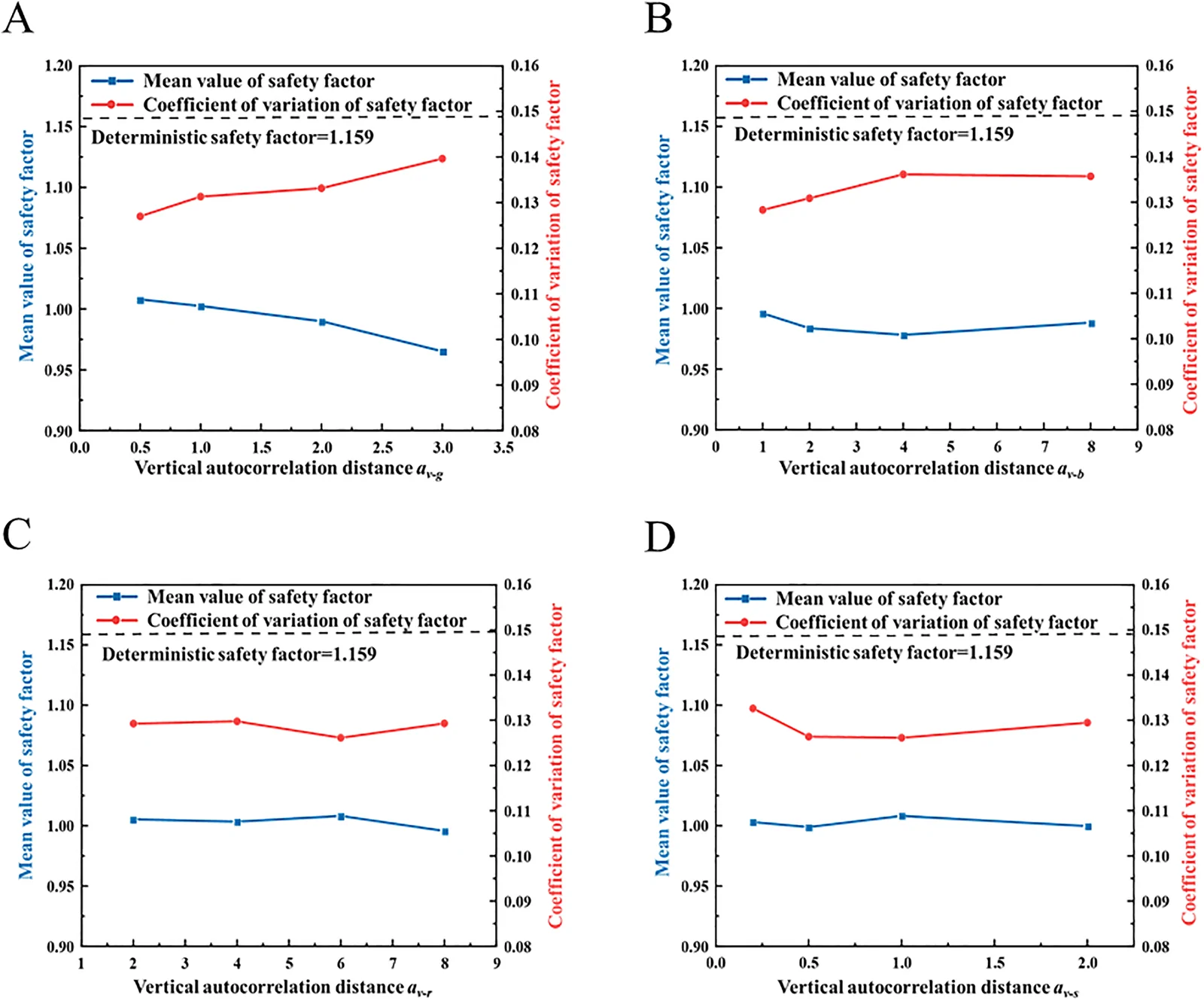
The influence of vertical autocorrelation distance on the mean value and COV of *F*_*s*_. (A) *R*_*g*_ zone. (B) *R*_*b*_ zone. (C) *R*_*r*_ zone. (D) *R*_*s*_ zone.

Fig 10 compares the variation patterns of the mean value and COV of the slope *F*_*s*_ under different vertical autocorrelation distances of the material strength parameters in the four random field zones. The *F*_s_ calculated based on the vertical autocorrelation distances of different random field zones are generally smaller than the deterministic calculation results. As the vertical autocorrelation distances of *R*_*g*_ and *R*_*b*_ increase, the mean value of *F*_*s*_ decreases, while the COV shows an increasing trend. The maximum mean value of *F*_*s*_ is 1.008, the minimum mean value is 0.965, the maximum COV is 0.140, and the minimum COV is 0.126. Variations in the vertical autocorrelation distances of the *R*_*r*_ and *R*_*s*_ zones have little effect on the mean value and COV of the slope *F*_*s*_. Overall, compared with the other three random field zones, the vertical autocorrelation distance of *R*_*g*_ has a more significant impact on slope stability. Furthermore, compared with the horizontal autocorrelation distance, variations in the vertical autocorrelation distance have a more pronounced effect on the *F*_*s*_. This is because the horizontal autocorrelation distance varies over a larger range, resulting in a larger corresponding variance reduction function value, whereas the variability of *φ* and *c* in the horizontal direction is reduced to a lesser extent.

**Fig 11:**
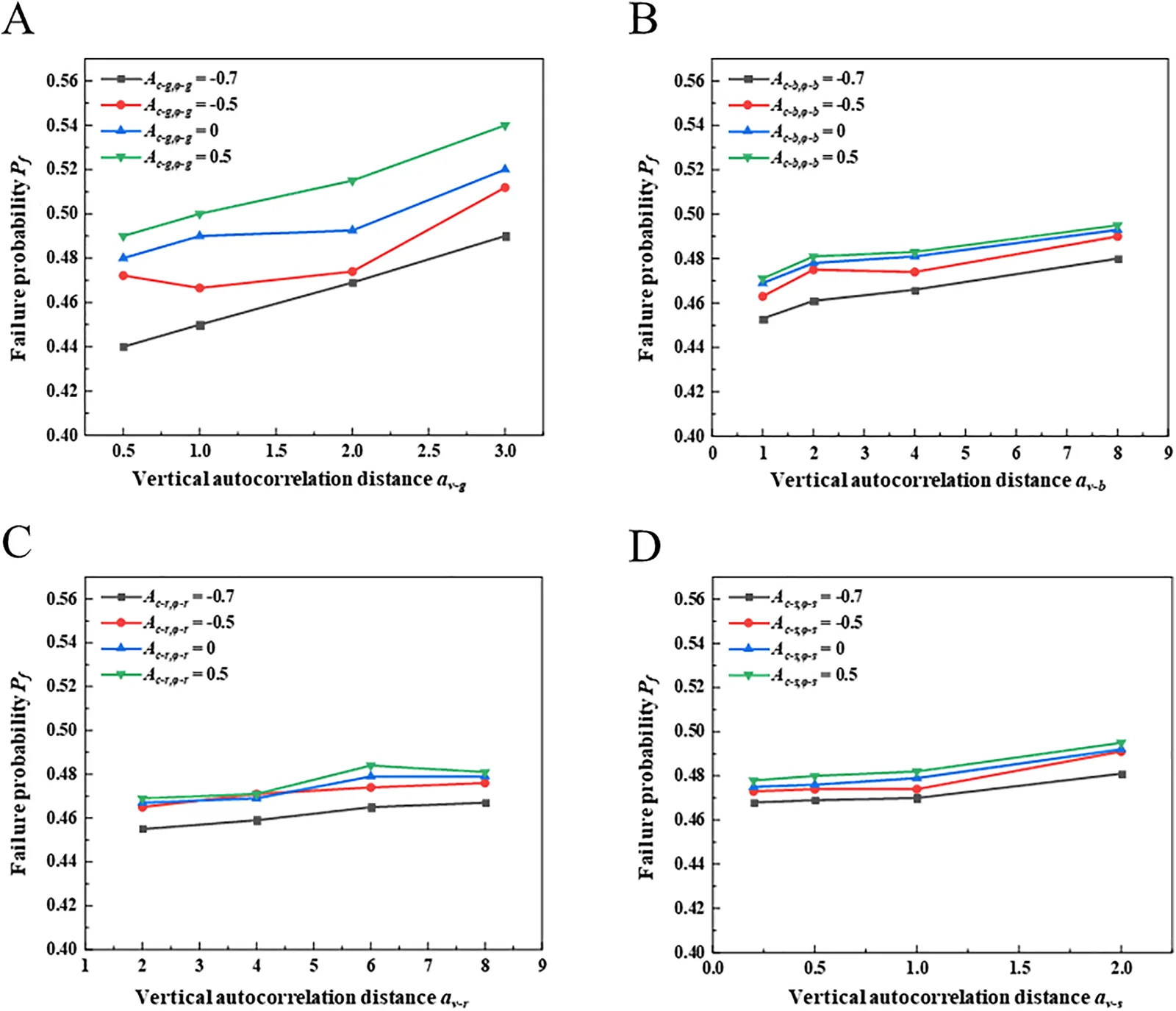
The influence of vertical autocorrelation distance on failure probability. (A) *R*_*g*_ zone. (B) *R*_*b*_ zone. (C) *R*_*r*_ zone. (D) *R*_*s*_ zone.

Fig 11 compares the influence of the vertical correlation distance of material strength parameters in different random field zones on the failure probability of the dam slope at the Tangjiashan landslide dam Reservoir. As the vertical correlation distance increases in the four random field zones, the failure probability of the dam slope increases accordingly. Across the range of vertical correlation distances considered, the maximum failure probability is 0.540, and the minimum is 0.440. Among the four zones, the failure probability of the dam slope increases at the highest rate with increasing vertical correlation distance in the *R*_*g*_ zone. The vertical correlation distance in the *R*_*b*_ zone has the second greatest influence on the growth rate of the failure probability, followed by the *R*_*r*_ zone and then the *R*_*s*_ zone. The influence of the vertical correlation distance in the four random field zones on the failure probability of the dam slope is consistent with that of the horizontal correlation distance. Furthermore, the failure probability of the dam slope is more sensitive to changes in the vertical correlation distance than to changes in the horizontal correlation distance.

#### 4.4.4. The influence of the COV of *c.*

**Fig 12:**
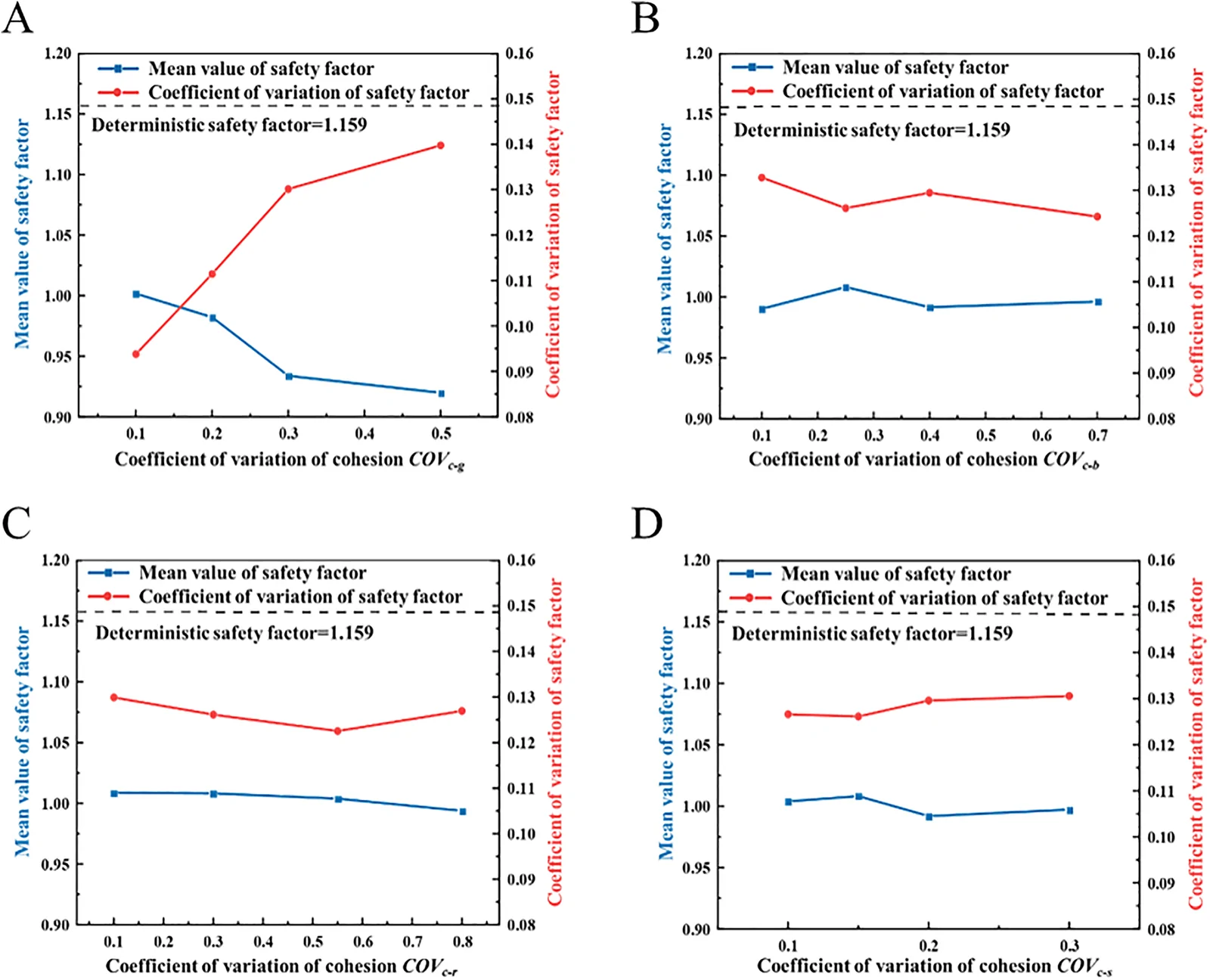
The influence of the COV of *c* on the mean value and the COV of *F*_*s*_. (A) *R*_*g*_ zone. (B) *R*_*b*_ zone. (C) *R*_*r*_ zone. (D) *R*_*s*_ zone.

The variation patterns of the mean value and COV of the slope *F*_*s*_ under different COVs of *c* are shown in Fig 12. Compared with the deterministic calculation results, the calculated slope *F*_*s*_ considering different random field zone COVs of *c* are all smaller. Across the considered COVs of *c*, the maximum mean value of *F*_*s*_ is 1.009, the minimum mean value of *F*_*s*_ is 0.920, the maximum COV of the *F*_*s*_ is 0.140, and the minimum COV is 0.094. As the COV of *c* of the material in the *R*_*g*_ zone increases, the mean value of *F*_*s*_ continuously decreases, while the COV continuously increases. When the COVs of *c* of the materials in the *R*_*b*_ and *R*_*s*_ zones increase, the mean value of *F*_*s*_ shows an overall decreasing trend, while the COV shows an overall increasing trend. In the *R*_*r*_ zone, an increase in the COV of *c* of the material leads to a slight decrease in both the mean value and COV of the slope *F*_*s*_. Overall, the influence of the COV of *c* of the material in the *R*_*g*_ zone on slope stability is the most significant, whereas changes in the COVs of *c* of the materials in the *R*_*r*_ and *R*_*s*_ zones have a relatively small impact. Furthermore, compared with the horizontal and vertical autocorrelation distances, variations in the *c* coefficient have a more significant impact on the slope *F*_*s*_.

**Fig 13:**
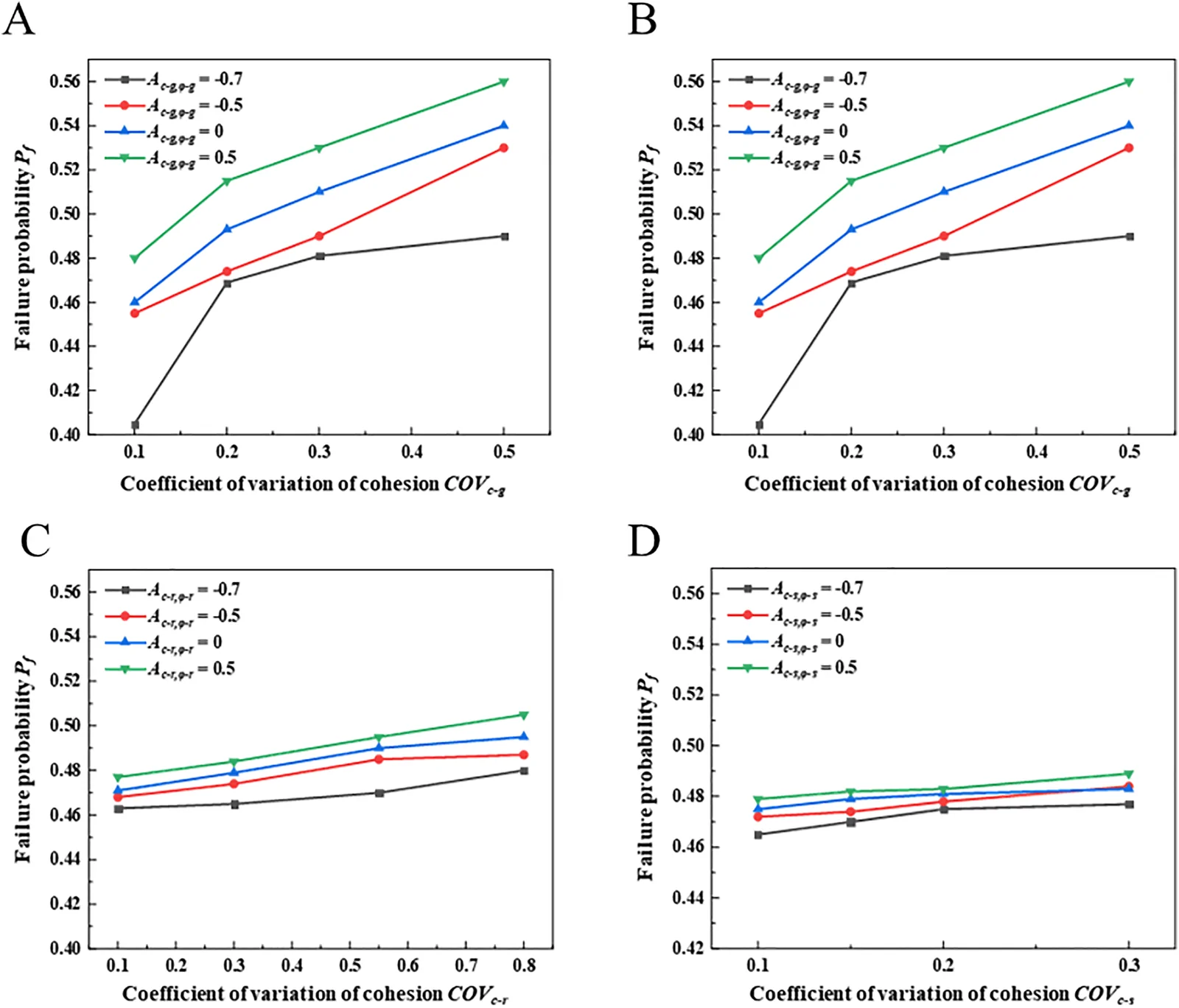
The influence of the COV of *c* on failure probability. (A) *R*_*g*_ zone. (B) *R*_*b*_ zone. (C) *R*_*r*_ zone. (D) *R*_*s*_ zone.

The comparison of the influence of the COV of *c* in different random field zones on the failure probability of the dam slope of the Tangjiashan landslide dam Reservoir is shown in Fig 13. Across the considered COVs of *c*, the maximum failure probability of the dam slope is 0.560, and the minimum is 0.405. As the COV of *c* of the materials in the four random field zones increases, the failure probability of the dam slope increases accordingly. Among the four zones, the *R*_*g*_ zone exhibits the most significant influence of the COV of *c* on the failure probability. When the COV of *c* is 0.1, the failure probability is 0.405; when it increases to 0.5, the failure probability rises to 0.490. The *R*_*b*_ zone has the second‑highest influence on the failure probability, following the zone. The *R*_*r*_ and *R*_*s*_ zones show negligible influences.

Compared with the horizontal and vertical autocorrelation distances, the failure probability of the dam slope is more sensitive to variations in the *c*. This is because the *c* in coarse-grained soil is primarily derived from frictional forces, also referred to as apparent *c* [24]. Consequently, the COV of *c* exerts a dominant influence on the failure probability of the dam slope through its effect on frictional forces — specifically, by affecting the internal structure and strength distribution of the soil.

#### 4.4.5. The influence of COV of *φ.*

**Fig 14:**
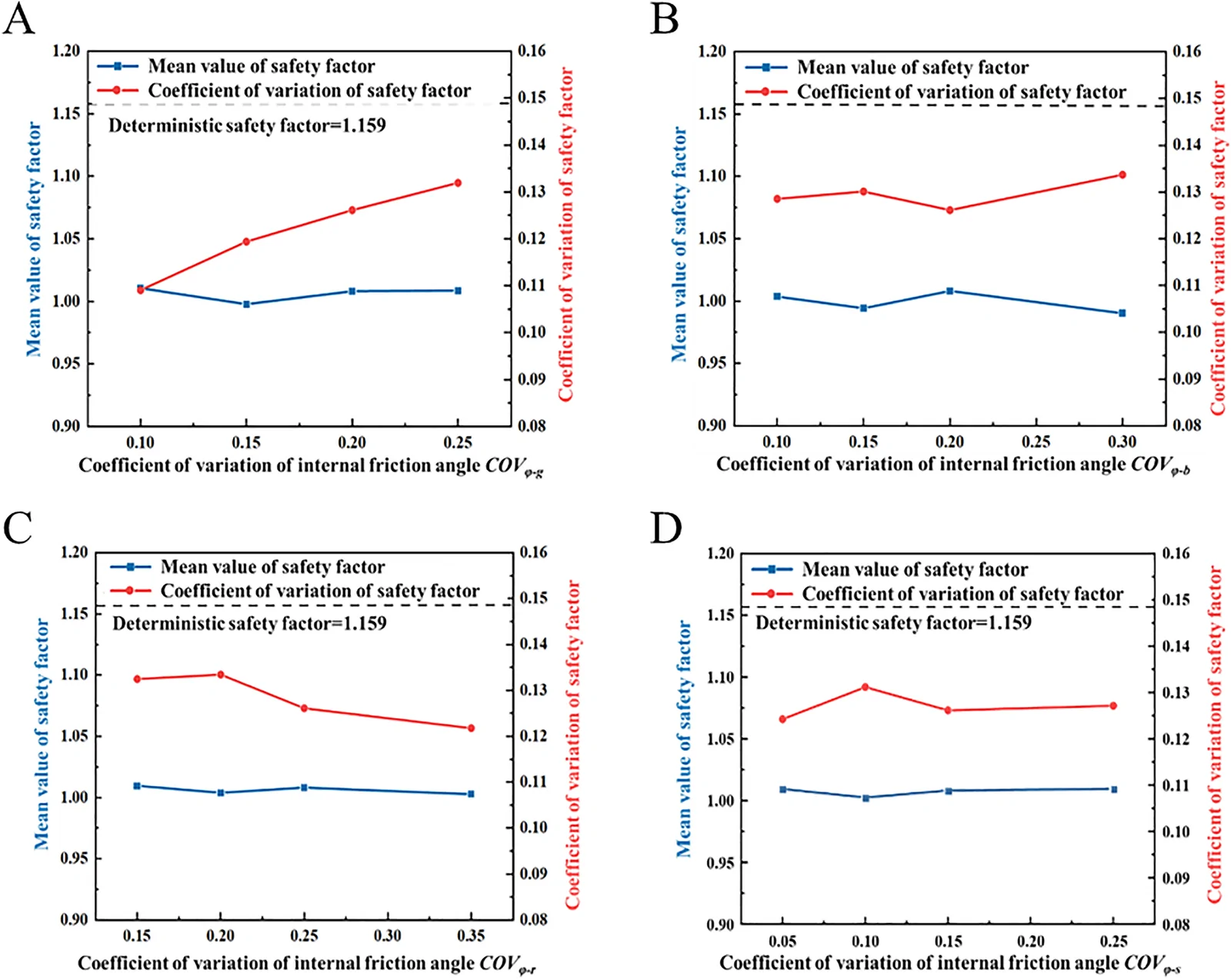
The influence of the COV of *φ* on the mean value and COV of *F*_*s*_. (A) *R*_*g*_ zone. (B) *R*_*b*_ zone. (C) *R*_*r*_ zone. (D) *R*_*s*_ zone.

Fig 14 presents the variation patterns of the mean value and COV of *F*_*s*_ under different COVs of *φ*. Compared with *F*_*s*_ calculated based on the COV of *φ* of the materials within different random field zones, the deterministic calculation results overestimate the stability of the dam slope. Across the considered COVs of *φ*, the maximum mean value of *F*_*s*_ is 1.011, the minimum mean value of *F*_*s*_ is 0.990, the maximum COV of the *F*_*s*_ is 0.134, and the minimum COV is 0.109. In the *R*_*g*_ zone, as the COV of *φ* increases, the COV of *F*_*s*_ continuously increases, while the mean value of *F*_*s*_ first decreases and then increases. In the *R*_*b*_ zone, as the COV of *φ* increases, the mean value of *F*_*s*_ exhibits a pattern of first decreasing, then increasing, and then decreasing again. In the *R*_*r*_ and *R*_*s*_ zones, as the COV of *φ* increases, the mean value of *F*_*s*_ remains largely unchanged, while the COV continuously decreases. Overall, the COV of *φ* has the greatest impact on *F*_*s*_ in the *R*_*g*_ zone, whereas the *R*_*r*_ and *R*_*s*_ zones are insensitive. Compared with the COV of *c*, the COV of *φ* has a smaller impact on the *F*_*s*_.

**Fig 15:**
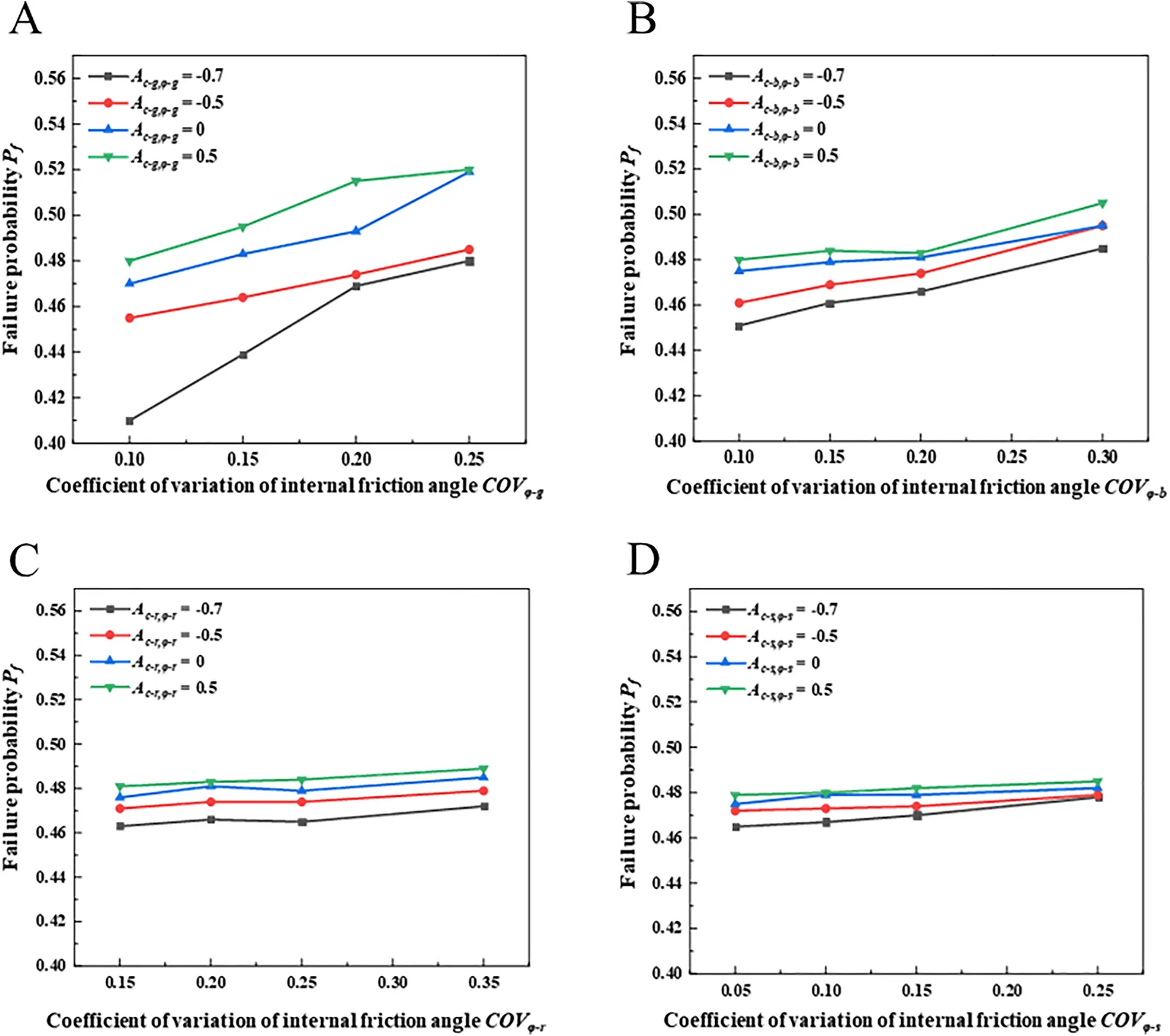
The influence of *COV* of *φ* on failure probability. (A) *R*_*g*_ zone. (B) *R*_*b*_ zone. (C) *R*_*r*_ zone. (D) *R*_*s*_ zone.

Fig 15 illustrates the influence of the COV of *c* in different random field zones on the failure probability of the dam slope. As shown in the Fig 15, an increase in the COV of *c* in the four random field zones leads to a corresponding increase in the failure probability of the dam slope. Across the considered COVs of *φ*, the maximum failure probability of the dam slope is 0.520, and the minimum is 0.410. Among the four zones, the COV of *c* in the *R*_*g*_ zone has the most significant influence on the failure probability, followed by the *R*_*b*_ zone. The COVs of *c* in the *R*_*r*_ and *R*_*s*_ zones have less pronounced effects on the stability of the dam slope. Overall, the influence of the variability of *φ* on the failure probability of the dam slope is smaller than that of the COV of *c*. Furthermore, compared with other influencing factors, the COV of *c* has the most prominent effect on the failure probability of the dam slope.

From the above analysis, it can be seen that variations in the material strength parameters in the *R*_*g*_ zone have the greatest impact on the failure probability of the dam slope, followed by the *R*_*b*_ zone. The influence of the COVs of *c* in the *R*_*r*_ and *R*_*s*_ zones is less pronounced. This is because the sliding surface of the dam slope at the Tangjiashan landslide dam is located in the *R*_*g*_ zone, and the *R*_*b*_ zone is adjacent to *R*_*g*_, whereas the *R*_*r*_ and *R*_*s*_ zones are relatively far from the *R*_*g*_ zone. Consequently, variations in the strength parameters of the materials in the *R*_*g*_ and *R*_*b*_ zones have the most significant impact on the stability of the dam slope.

#### 4.4.6. The influence of the cross-correlation coefficient.

**Fig 16:**
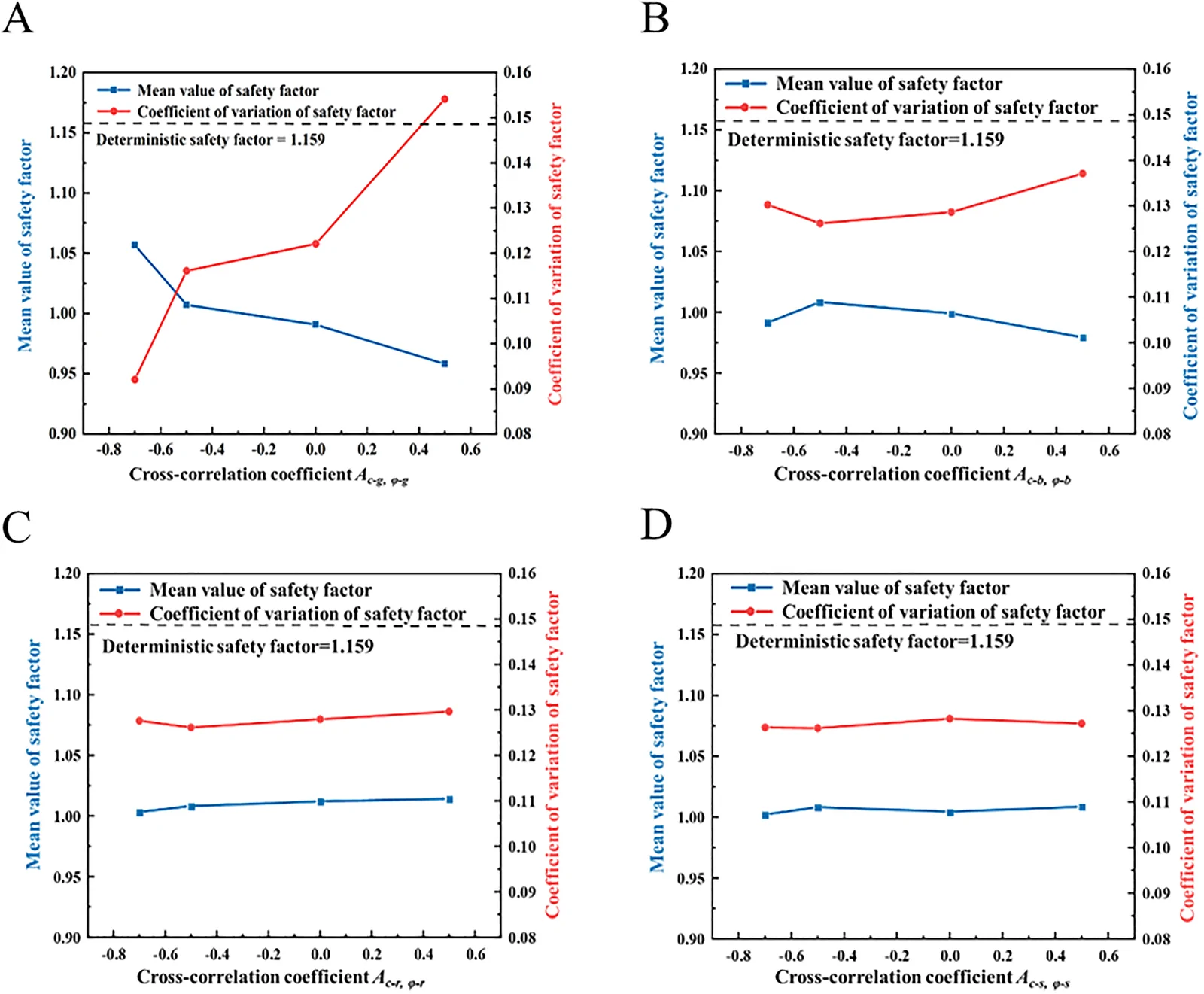
The influence of the cross-correlation coefficient on the mean value and COV of the *F*_*s*_. (A) *R*_*g*_ zone. (B) *R*_*b*_ zone. (C) *R*_*r*_ zone. (D) *R*_*s*_ zone.

Fig 16 compares the variation patterns of the mean value and COV of *F*_*s*_ under different correlation coefficients between the shear strength parameters *c* and *φ* of the materials in the random field zones. The maximum *F*_*s*_ calculated based on the correlation coefficient between *c* and *φ* is 1.057, which is lower than the deterministic calculation result. By synthesizing the dam slope stability calculation results from Sections 4.4.2 to 4.4.6, it is found that the *F*_*s*_ of the Tangjiashan landslide dam slope calculated using random field theory is consistently smaller than that obtained from deterministic calculations.

Overall, the cross-correlation coefficient between *c* and *φ* in *R*_*g*_ zone has the most significant impact on the mean value and COV of *F*_*s*_. Specifically, the larger the cross-correlation coefficient, the smaller the mean value of *F*_*s*_ and the larger the COV of *F*_*s*_. As the cross-correlation coefficient between *c* and *φ* in *R*_*b*_ zone increases, the mean value of *F*_*s*_ shows a trend of increasing slightly first and then decreasing, while the COV shows a trend of decreasing slightly first and then increasing. The cross-correlation coefficients between *c* and *φ* in *R*_*r*_ and *R*_*s*_ zone have no significant impact on the *F*_*s*_. Compared with the horizontal autocorrelation distance, vertical autocorrelation distance, COV of *c*, and COV of *φ*, the cross-correlation coefficient has the most significant impact on the *F*_*s*_.

#### 4.4.7. The distribution of critical slip surfaces.

**Fig 17:**
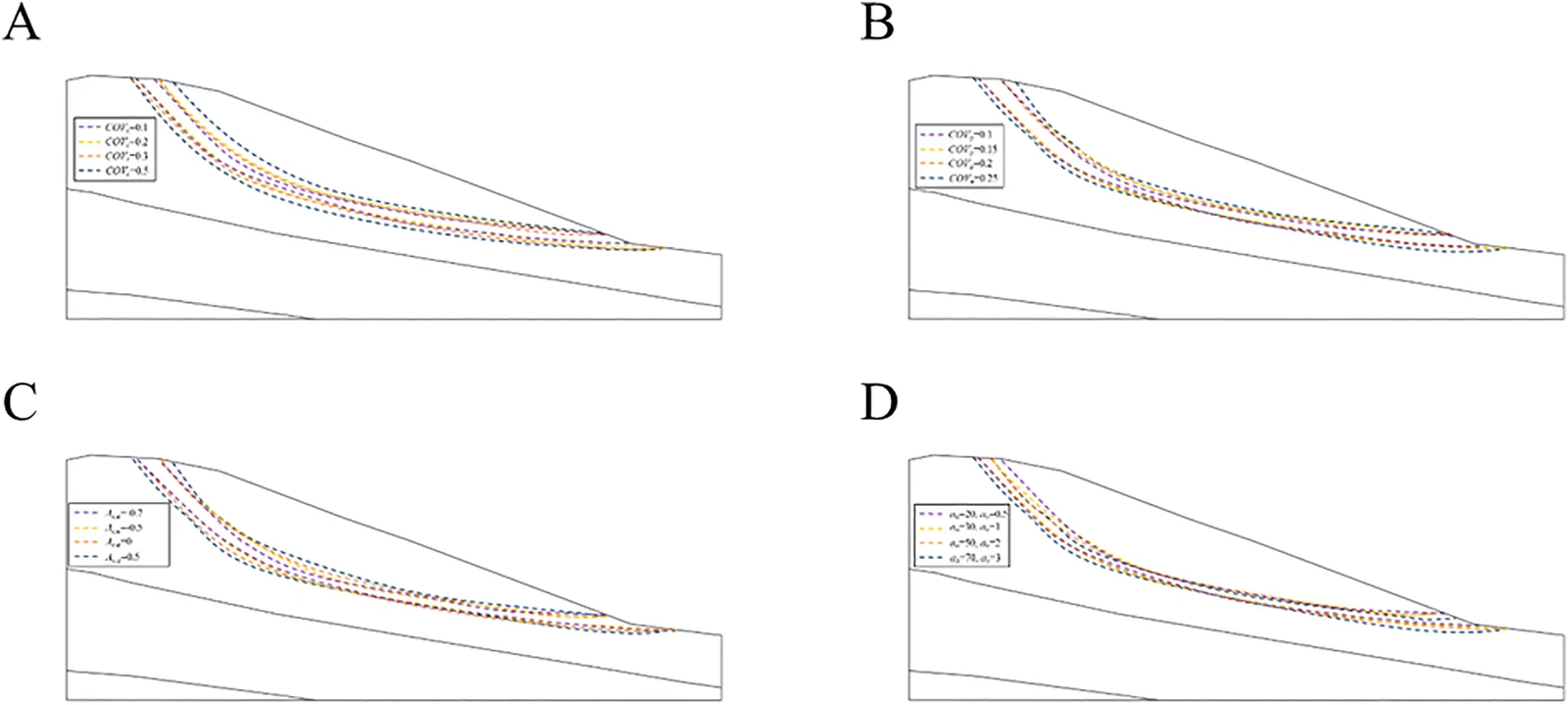
Slip surface distribution under different random field parameters. (A) Slip surface distribution under different *COV*_*c*_. (B) Slip surface distribution under different *COV*_*φ*_. (C) Slip surface distribution under different cross-correlation coefficient. (D) Slip surface distribution under different autocorrelation distance.

To reveal the variability of failure mechanisms under different random field conditions, we extracted the critical slip surfaces from all stochastic realizations and constructed envelope diagrams showing the upper and lower bounds of slip surface locations. Fig 17 presents the envelopes of critical slip surfaces obtained under varying coefficients of variation of cohesion (*COV*_*c*_), coefficients of variation of friction angle (*COV*_*φ*_), cross-correlation coefficients between *c* and *φ*, and autocorrelation distances, based on 500 Monte Carlo simulations for each parameter combination, superimposed on the deterministic slip surface for comparison.

For each parameter combination, the two slip surfaces represented by the same color in Fig 17 denote the upper bound (the shallowest slip surface) and the lower bound (the deepest slip surface) among all stochastic slip surfaces, respectively. The region between these two bounds represents the full range of possible slip surface locations under the given random field conditions. The results show that, across all random field parameter combinations considered, the stochastic slip surfaces remain entirely within the gravel soil zone (*R*_*g*_) and also fall within the sliding zone identified by the deterministic analysis. This confirms that the sliding zone determined deterministically is robust and sufficiently encompasses the potential failure mechanisms induced by spatial variability.

Further analysis reveals that the influence of the aforementioned random field parameters on slip surface distribution can be summarized in two aspects. The first concerns the effects of *COV*_*c*_, *COV*_*φ*_, and the cross-correlation coefficient between *c* and *φ*. As these parameters increase, the width of the slip surface envelope expands, indicating that the potential slip surfaces tend to be more dispersed. Specifically, the upper bound of the envelope (i.e., the shallowest slip surface) moves upward, accompanied by a reduction in the minimum slip surface area; meanwhile, the lower bound (i.e., the deepest slip surface) moves downward, with a corresponding increase in the maximum slip surface area. This implies that under higher parameter variability, local weak zones are more likely to form within the slope, making the actual location of the critical slip surface more sensitive to specific random field realizations. Consequently, the overall distribution of potential sliding zones becomes broader, and the geometry of the slip surfaces exhibits greater scatter across different realizations. Among these three factors, *COV*_*c*_ exerts the most significant influence on the dispersion of slip surfaces.

The second pertains to the influence of the autocorrelation distance. As the autocorrelation distance increases, the overall position of the slip surface shifts downward, meaning that the critical slip surface tends to develop at greater depths. This is because a larger autocorrelation distance smooths the spatial fluctuations of the strength parameters, reducing the likelihood of localized weak zones in the shallow subsurface. As a result, the critical slip surface is forced to seek a failure path at greater depths where the strength is relatively more uniform.

## 5. Dam slope stability analysis based on local random field model

### 5.1. The establishment of local random field model

In slope stability research using the strength reduction method, some scholars have found that slope instability is caused by the reduction of local soil strength. Consequently, they proposed a local strength reduction method that reduces the strength of only local soil units while keeping the original strength of other soil units unchanged [25]. The deterioration process of strength parameters in rock and soil slopes is not an overall process but rather an incremental one, progressing from local damage to overall instability. In natural rock and soil slopes, special structural planes such as joints, bedding planes, and weak structural layers are commonly present, and slope instability or landslides often occur along such planes. Therefore, it is particularly important to perform slope strength reduction specifically on weak structural planes. The core of the local strength reduction method lies in reducing the mechanical parameters of the rock and soil in the critical zone (i.e., the sliding surface zone), thereby obtaining the *F*_*s*_ and displacement of the slope. Yang et al. [18] calculated the *F*_*s*_ and displacement using the local strength reduction method and found that the resulting values are smaller than those obtained from the overall strength reduction method, which are more consistent with the actual stability of the slope. They concluded that applying the local strength reduction method to heterogeneous slopes is more reasonable than using the overall strength reduction method. Dai et al. [25] used the strength reduction method to identify the sliding surface soil zone and established a corresponding slope model using particle flow software. They achieved local strength reduction by reducing the strength of the soil in the sliding surface zone and suggested that this method provides greater accuracy for slope stability analysis.

The overall mesh of the Tangjiashan landslide dam slope is relatively fine, resulting in an excessive number of random field realizations when using the global random field model, which in turn leads to high computational costs. Given that some researchers have adopted the local strength reduction method to compute slope *F*_*s*_, and that its validity and feasibility have been demonstrated [16], this chapter establishes a local random field model for strength parameters. The model is used to investigate the influence of the randomness of material strength parameters within the sliding surface zone on the stability of the Tangjiashan landslide dam slope. Furthermore, the results and computational efficiency of the local random field model are compared with those of the global random field model to verify the feasibility and efficiency of the proposed local approach.

**Fig 18:**
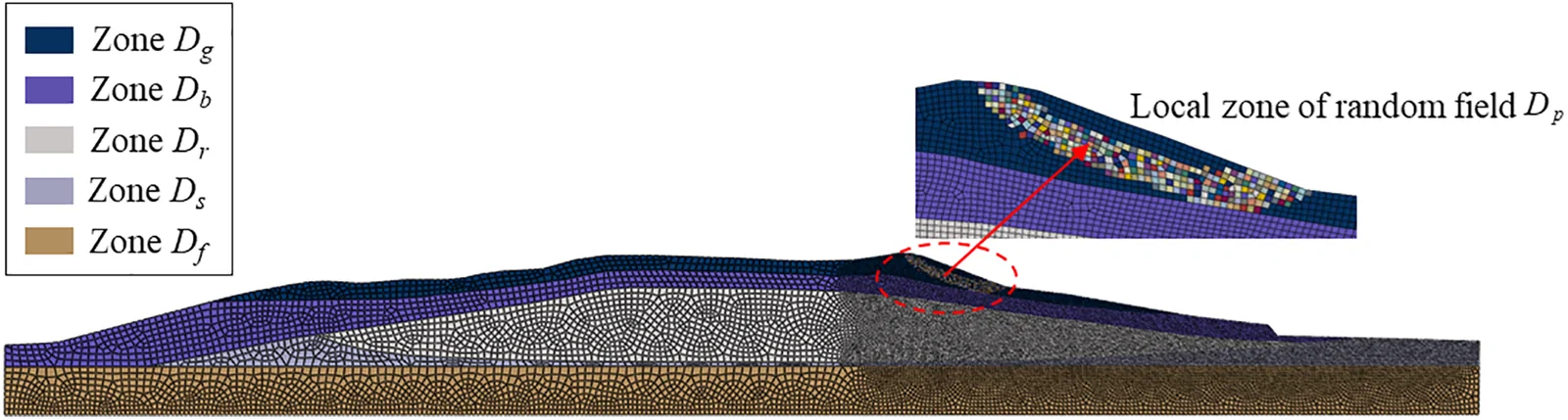
Selection of local random field zone.

According to the analysis in Section 4.4.7, the sliding surfaces of the Tangjiashan landslide dam under the global random field model are all within the calculation area of the deterministic stability sliding surface. Therefore, this sliding surface area is identified as a local random field area, denoted as *D*_*p*_. As shown in Fig 18, this local random field zone contains a total of 283 elements.

Based on the analysis in Section 3, and given that all elements of the sliding surface zone are located within the gravel soil *D*_*g*_ zone, this section determines and combines the material strength parameters in sliding surface zone using the strength parameters of the gravel soil *D*_*g*_ zone as a basis. The specific parameter combination is presented in Table 3.

**Table 3:** Parameter combination of *D*_*p*_ sliding surface zone.

Combination	1	2	3	4	5	6	7	8	9	10	11	12	13
***a***_***h-p***_	**50**	**20**	**30**	**70**	50	50	50	50	50	50	50	50	50
***a***_***v-p***_	**2**	2	2	2	**0.5**	**1**	**3**	2	2	2	2	2	2
***COV***_***c-p***_	**0.2**	0.3	0.3	0.3	0.3	0.3	0.3	**0.1**	**0.3**	**0.5**	0.3	0.3	0.3
***COV***_***φ-p***_	**0.1**	0.2	0.2	0.2	0.2	0.2	0.2	0.2	0.2	0.2	**0.15**	**0.2**	**0.25**
***A***_***c-p, φ-p***_	−0.7, **−0.5**, 0, 0.5												

**Fig 19:**
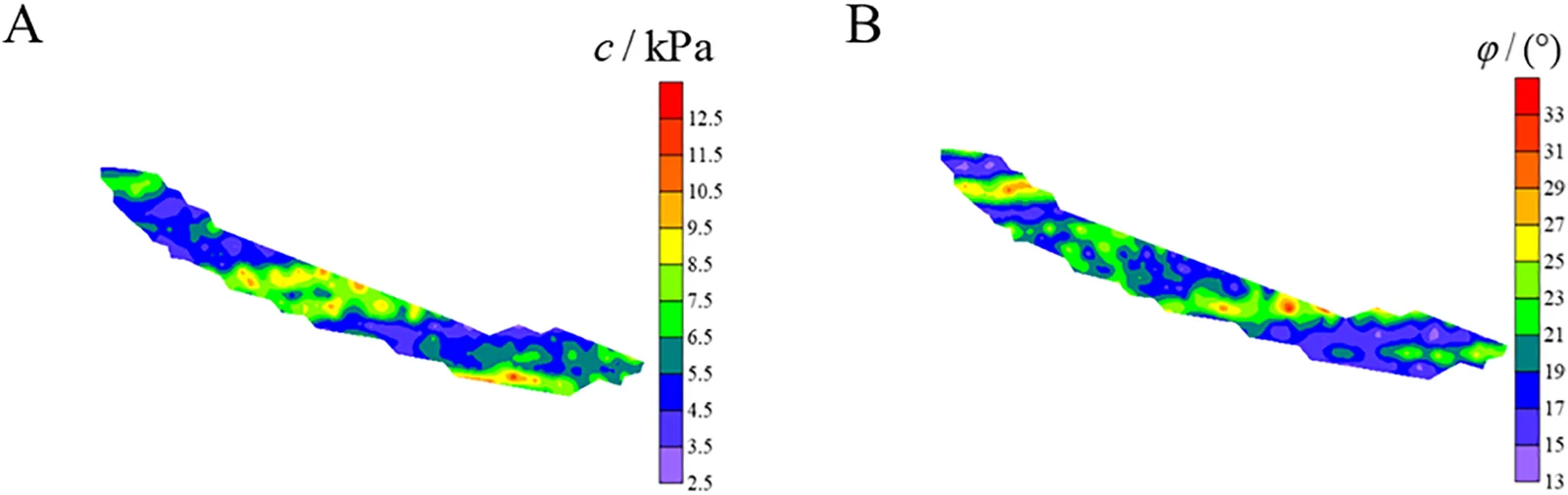
A typical realization of *c* and *φ* based on the local random field model. (A) *c*. (B). *φ*.

Fig 19 takes combination 1 in Table 3 as an example and plots the cloud diagrams of the shear strength parameters *c* and *φ* in the sliding surface zone under a typical realization of the local random field model. It can be observed that both the local random parameters *c* and *φ* and their cloud diagrams exhibit certain horizontal banding characteristics. This is mainly because the horizontal autocorrelation distances of *c* and *φ* are greater than their vertical autocorrelation distances. Furthermore, since the cross-correlation coefficient between *c* and *φ* in this typical realization of the local random field model is −0.5, as expected, *c* and *φ* in Fig 19 also show a certain negative correlation.

### 5.2. Convergence analysis of Monte Carlo simulation results

**Fig 20:**
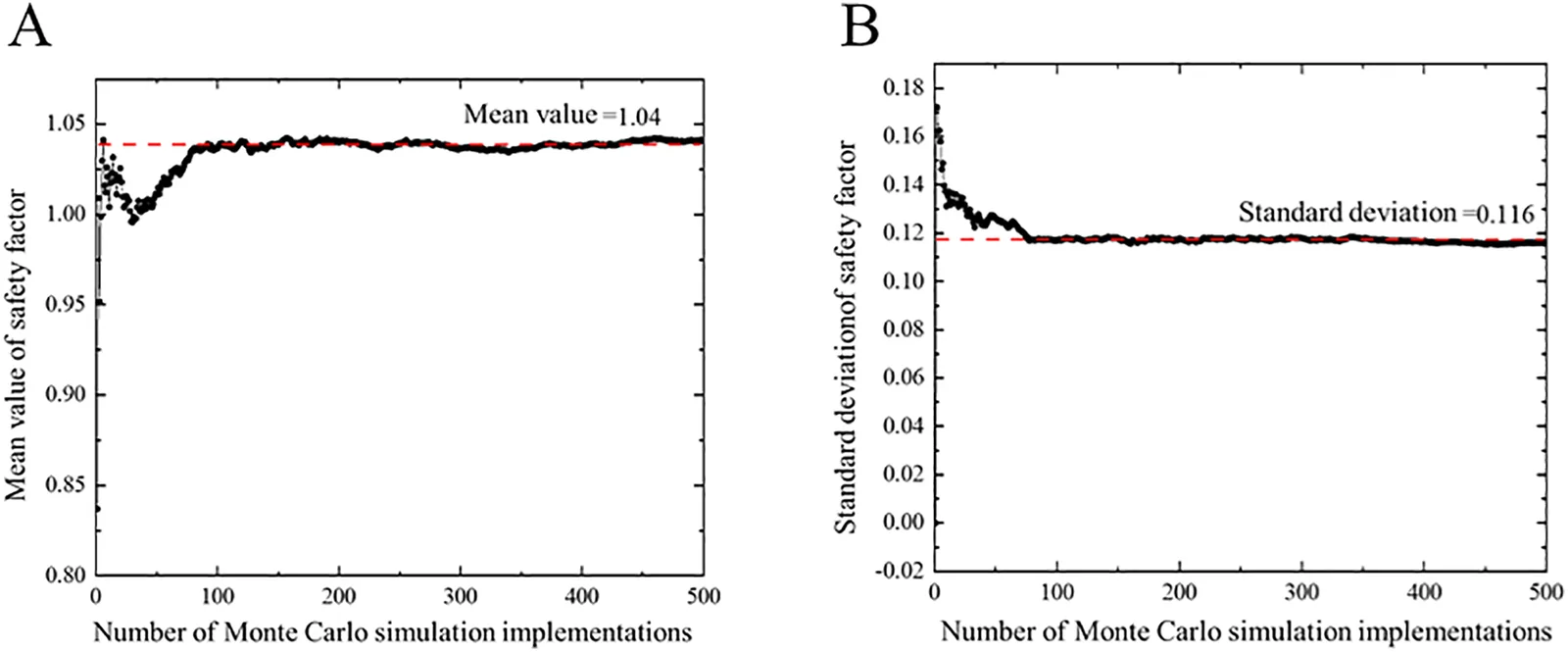
Convergence curve of *F*_*s*_ of landslide dam slope based on local random field model. (A) Convergence curve of mean value of *F*_*s*_. (B) Convergence curve of standard deviation of *F*_*s*_.

To obtain stable estimates of the *F*_*s*_, 500 Monte Carlo simulations are performed for all parameter combinations of the local conditional random field model (Table 3), as shown in Fig 20. Fig 20A and 20B present the mean value and standard deviation of the *F*_*s*_, respectively, calculated from 500 Monte Carlo simulations under combination 1. It can be seen from Fig 20 that 100 simulations are sufficient to achieve adequate convergence of the *F*_*s*_. The convergence behavior for other combinations is similar to that observed for combination 1. Therefore, in the subsequent analysis, 100 random simulations of material strength parameters will be adopted for the sliding surface zone elements in combinations 1–13, ensuring sufficiently reliable results while minimizing computational effort.

### 5.3. Results

#### 5.3.1. Analysis of the stability of the dam slope.

**Fig 21:**
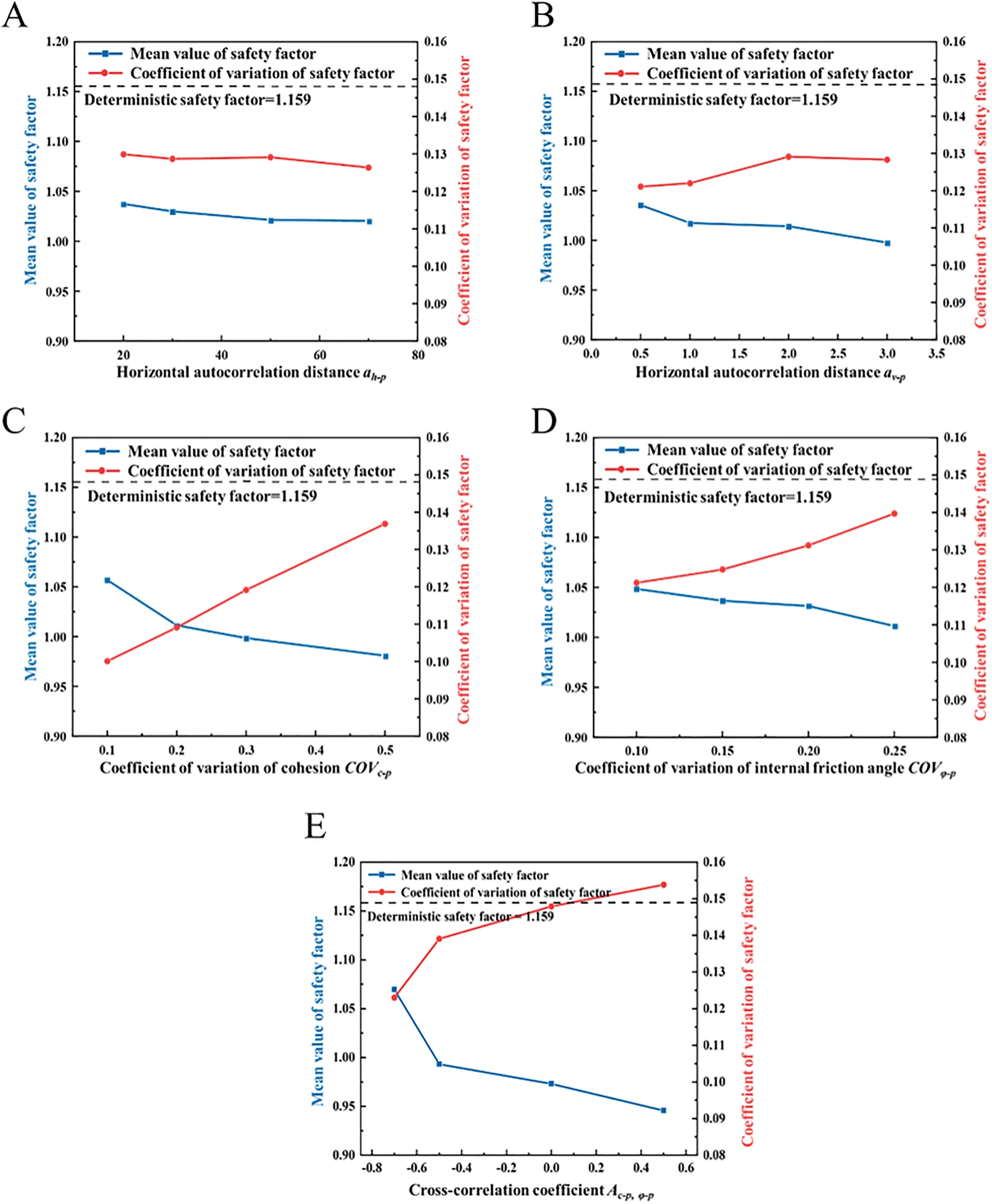
The influence of each parameter on the mean value and COV of the *F*_*s*_. (A) Horizontal autocorrelation distance. (B) Vertical autocorrelation distance. (C). COV of *c.* (D) COV of *φ.* (E) Cross-correlation coefficient.

For a concise analysis, this section examines the mean value and COV of *F*_*s*_ calculated with a cross-correlation coefficient of –0.5. Fig 21 illustrates how the mean value and COV of *F*_*s*_, computed using the local random field model, vary with increases in the horizontal autocorrelation distance, vertical autocorrelation distance, COV of *c*, COV of *φ*, and cross-correlation coefficient. As shown in Fig 21, considering the effects of different horizontal autocorrelation distances, vertical autocorrelation distances, COVs of *c* and *φ*, and cross-correlation coefficients, the maximum *F*_*s*_ calculated using the local random field model is 1.070, and the minimum is 0.950. Both values are smaller than the deterministic calculation results. Comparing these results with those from Section 4.4, the *F*_*s*_ obtained from the local random field model and the global random field model are found to be close. In the local random field model, as the horizontal autocorrelation distance increases, the mean value of *F*_*s*_ decreases slightly, while the COV of the *F*_*s*_ remains essentially unchanged. As the vertical autocorrelation distance, COV of *c*, COV of *φ*, and cross-correlation coefficient between *c* and *φ* in *D*_*p*_ zone increase, the mean value of *F*_*s*_ shows a decreasing trend, and the COV continuously increases. Among these factors, the cross-correlation coefficient and the COV of *c* have the most significant impact on the *F*_*s*_, followed by the COV of *φ* and the vertical autocorrelation distance, whereas the horizontal autocorrelation distance has a less pronounced effect. Compared with the analysis results in Section 4.4, the variation patterns of the mean value of *F*_*s*_ and the COV derived from the local random field model are consistent with those from the global random field model.

**Fig 22:**
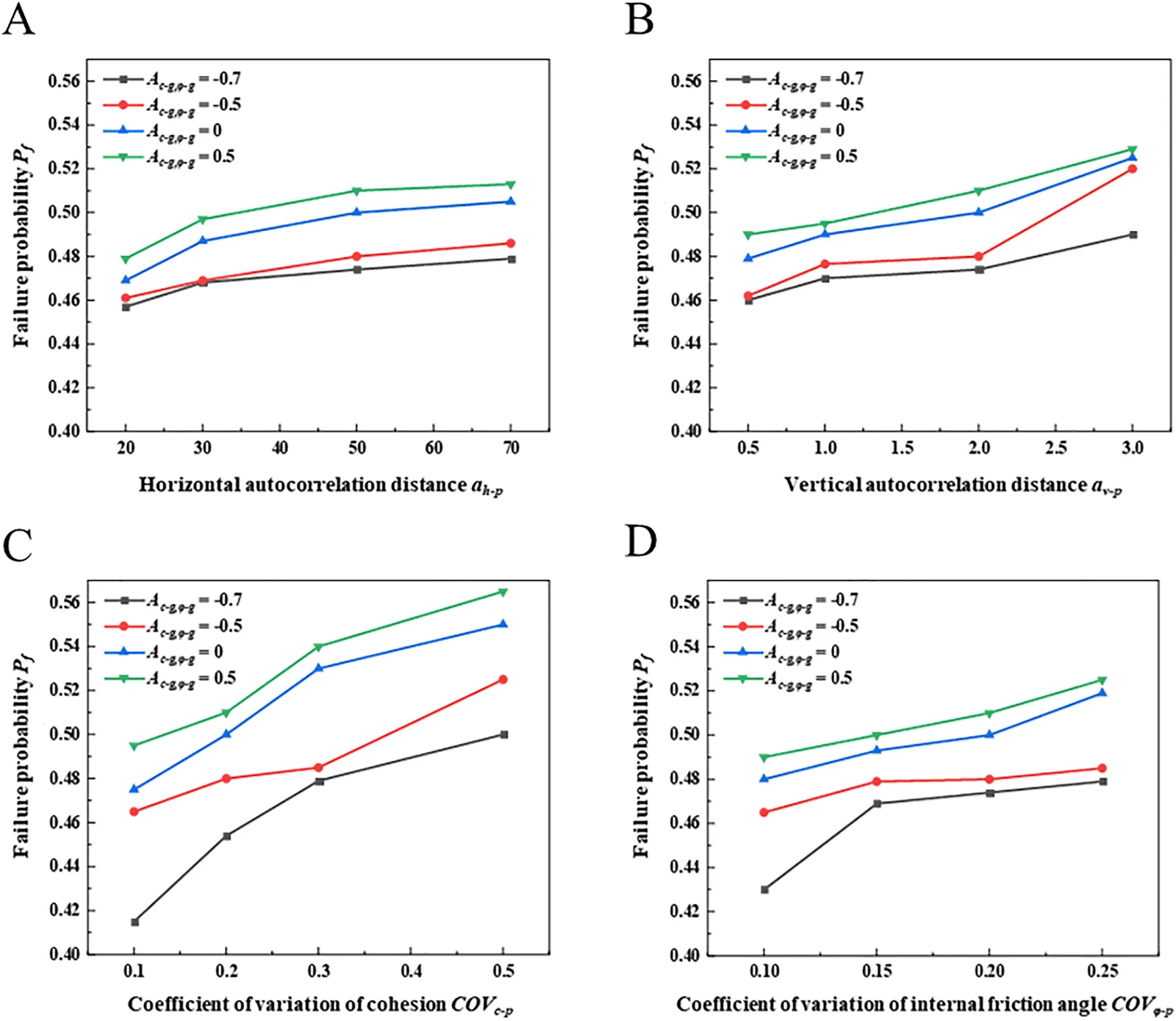
The influence of each parameter on the failure probability. (A) Horizontal autocorrelation distance. (B) Vertical autocorrelation distance. (C). COV of *c.* (D) COV of *φ.*

Fig 22 illustrates how the failure probability of the dam slope varies with the horizontal autocorrelation distance, vertical autocorrelation distance, COV of *c*, COV of *φ*, and cross-correlation coefficient. The calculated maximum failure probability is 0.565, and the minimum is 0.415. Compared with the global random field model, the local random field model yields a larger maximum failure probability. As shown in Fig 22, the failure probability increases with both the horizontal and vertical autocorrelation distances, with the vertical distance having a more significant influence than the horizontal one. The larger the cross-correlation coefficient between *c* and *φ*, the higher the failure probability and the worse the dam slope stability. The failure probability also increases with the COVs of *c* and *φ*, and it is most sensitive to changes in *c*. A comprehensive comparison with the analysis results in Section 4.4 shows that the variation pattern of the failure probability obtained from the local random field model is consistent with that from the global random field model.

#### 5.3.2. Comparison and analysis of computational efficiency with the global random field model.

In Section 4.4, when performing calculations using the global random field model for the Tangjiashan landslide dam, the parameters of the four random field zones (*R*_*g*_, *R*_*b*_, *R*_*r*_, and *R*_*s*_) need to be randomly arranged and combined, resulting in a total of 196 global random field models. For the global random field model, each of the 196 parameter combinations required 500 simulations. Generating the 500 input files for one combination took ~13 minutes, and solving them on a six-core computer took ~32 hours. Thus, the total computational time for all 196 combinations, running one job at a time, would be approximately 6,300 hours (32 hours × 196). In contrast, the local random field model required only 52 combinations and 100 simulations per combination, reducing the total time to approximately 234 hours—an improvement in efficiency of nearly two orders of magnitude.

When generating local random field models, only the material strength parameters of the sliding surface zone need to be randomly varied. Each local random field zone contains only 283 grid cells, and these cells are located exclusively within the *D*_*g*_ zone. The material homogeneity of these grid cells simplifies the arrangement and combination of parameters across different zones, requiring only 52 random field model calculations for the sliding surface *D*_*p*_ zone. One operation using the local random field model requires generating 100 input files, which takes only about 6 seconds. Executing the 100 calculation tasks on a six-core computer takes approximately 4.5 hours. If only one job is run at a time, the total computation time for the *F*_*s*_ analysis based on the local random field model is approximately 234 hours.

Overall, compared with the global random field model, using the local random field model to study the influence of spatial variability of the Tangjiashan landslide dam materials not only yields accurate calculation results but also significantly improves computational efficiency. Therefore, when the sliding surface zone is located within a single material zone, this chapter suggests that the local random field model can be used for dam slope stability analysis considering the variability of material strength parameters, facilitating accurate and rapid assessment of dam slope stability.

## 6. Conclusion

This paper adopts random field theory and takes the Tangjiashan landslide dam as the research object. A global random field model considering the cross-correlation of material strength parameters on the dam slope is established. The *F*_*s*_ and failure probability of the dam slope are calculated, and a parameter sensitivity analysis is conducted. To enable efficient calculation and analysis of dam slope stability considering the variability of material strength parameters, a local random field model is further developed. The calculation results and computational efficiency of the local random field model are compared with those of the global random field model to verify the feasibility and efficiency of the local approach. The main conclusions are as follows:

(1) The variability of material strength parameters in the *R*_*g*_ zone has the most significant impact on the *F*_*s*_ of the dam slope, followed by the *R*_*b*_ zone, with the *R*_*s*_ zone having the smallest influence. The *F*_*s*_ calculated using the random field model is smaller than the deterministic calculation result, indicating that ignoring the variability of material strength parameters will overestimate the stability of the dam slope.
(2) As the vertical autocorrelation distance, COV of *c*, and cross-correlation coefficient of the material strength parameters in the *R*_*g*_ zone increase, the mean value of *F*_*s*_ continuously decreases, while the COV of *F*_*s*_ continuously increases. Among these factors, the cross-correlation coefficient has the most significant impact on the *F*_*s*_. The horizontal autocorrelation distance and the variability of *φ* in the *R*_*g*_ zone have a relatively small influence on the mean value and COV of *F*_*s*_.
(3) An increase in the horizontal and vertical autocorrelation distances, COV of *c*, COV of *φ*, and cross-correlation coefficient of the material strength parameters in the four random field zones all lead to an increase in the failure probability of the dam slope. The failure probability is most sensitive to the COV of *c*. Compared with the other random field zones, variations in material strength parameters in the *R*_*g*_ zone have the most significant impact on the failure probability of the dam slope.
(4) The dam slope stability analysis based on the local random field model yields the same trends as those based on the global random field model, while the local model offers higher computational efficiency. Therefore, for practical engineering applications, when the sliding surface zone is located within a single material partition, the local random field model is recommended, as it facilitates accurate and rapid analysis of dam slope stability considering the variability of material strength parameters.

## 7. Limitations and future work

Although this study provides a preliminary framework for the stability analysis of landslide dam slopes considering the spatial variability of material parameters, several limitations should be acknowledged.

(1) The actual landslide dam is a three-dimensional geological body, and two-dimensional analysis cannot fully capture lateral topographic variations and lateral confinement effects [26]. Previous studies have shown that 2D analysis typically neglects the shear resistance along the lateral boundaries of the sliding mass (i.e., the end effect) and tends to yield lower safety factors and underestimate runout distance compared with 3D analysis in limit equilibrium assessments [27]. However, given the time urgency of emergency response for landslide dams, the 2D model can significantly reduce computation time and is therefore practically feasible for this study. For projects with complex topographic conditions or higher importance, more refined 3D analysis is still recommended.
(2) Due to data scarcity, the autocorrelation distances and other random field parameters listed in Table 2 are mainly based on literature values rather than in-situ measurements from the Tangjiashan landslide dam. More accurate results would require future site-specific investigations and laboratory tests to better calibrate these statistical parameters.
(3) This study simulates the impoundment process through 11 analysis steps, but within each step the dam is assumed to be under hydrostatic conditions and fully saturated, without considering seepage processes. The stability of the dam slope may therefore be overestimated. Fully coupled hydromechanical analysis can more accurately characterize pore pressure evolution and effective stress changes under transient seepage conditions, but at a substantially higher computational cost. Therefore, the stability assessment results presented herein should be regarded as preliminary estimates under long-term saturated conditions.

In future work, we will attempt to: (1) extend the analysis to three dimensions to more comprehensively evaluate topographic and lateral variability effects; (2) conduct targeted site investigations to better calibrate the random field parameters; (3) establish coupled hydromechanical models incorporating seepage effects; and (4) explore more efficient stochastic simulation methods to reduce the computational cost of 3D or coupled analyses.
